# Endogenous pore-forming protein complex targets acidic glycosphingolipids in lipid rafts to initiate endolysosome regulation

**DOI:** 10.1038/s42003-019-0304-y

**Published:** 2019-02-11

**Authors:** Xiao-Long Guo, Ling-Zhen Liu, Qi-Quan Wang, Jin-Yang Liang, Wen-Hui Lee, Yang Xiang, Sheng-An Li, Yun Zhang

**Affiliations:** 1 Key Laboratory of Animal Models and Human Disease Mechanisms of The Chinese Academy of Sciences/Key Laboratory of Bioactive Peptides of Yunnan Province, Kunming Institute of Zoology, the Chinese Academy of Sciences, Kunming, Yunnan 650223 China; 2 Kunming College of Life Science, University of Chinese Academy of Sciences, Kunming, Yunnan 650204 China; 3 Center for Excellence in Animal Evolution and Genetics, Chinese Academy of Sciences, Kunming, Yunnan 650223 China

## Abstract

Bacterial pore-forming toxin aerolysin-like proteins (ALPs) are widely distributed in animals and plants. However, functional studies on these ALPs remain in their infancy. βγ-CAT is the first example of a secreted pore-forming protein that functions to modulate the endolysosome pathway via endocytosis and pore formation on endolysosomes. However, the specific cell surface molecules mediating the action of βγ-CAT remain elusive. Here, the actions of βγ-CAT were largely attenuated by either addition or elimination of acidic glycosphingolipids (AGSLs). Further study revealed that the ALP and trefoil factor (TFF) subunits of βγ-CAT bind to gangliosides and sulfatides, respectively. Additionally, disruption of lipid rafts largely impaired the actions of βγ-CAT. Finally, the ability of βγ-CAT to clear pathogens was attenuated in AGSL-eliminated frogs. These findings revealed a previously unknown double binding pattern of an animal-secreted ALP in complex with TFF that initiates ALP-induced endolysosomal pathway regulation, ultimately leading to effective antimicrobial responses.

## Abstract (web summary)

Guo et al. identify the role of acidic glycosphingolipids as the mechanism of action of the pore-forming protein complex βγ-CAT from the frog. This study suggests that βγ-CAT binds to both gangliosides and sulfatides, initiating its endocytosis and ultimately exerting its antimicrobial effects.

## Introduction

Cellular membranes are essential for defining the boundary and maintaining the compartmentalization of living cells. After synthesis in ribosomes, classical membrane receptors, ion channels and transporters are integrated into defined cellular membranes. Pore-forming proteins are usually secreted proteins and exist in a water-soluble monomeric form^1,2^. After undergoing an extensive conformational change under specific conditions, these nonclassical membrane proteins can form transmembrane pores of various sizes (2 to 50 nm), which function as channels for passing different molecules, including ions, proteins, peptides and nucleic acids^3–5^. In addition to their well-known functions in cell death^6,7^, emerging evidence suggests that pore-forming proteins play pivotal pathophysiological roles in living organisms, functioning in processes such as cell differentiation, reproduction and tissue repair^3,8–10^, but the related mechanisms remain unclear.

Aerolysins are a type of bacterial β-barrel pore-forming toxin belonging to a specific type of pore-forming proteins produced by *Aeromonas* species^11^. Interestingly, proteins with an aerolysin membrane insertion domain, named aerolysin-like proteins (ALPs), exist widely in animals and plants^12,13^, and evidence concerning their pivotal roles in animal and plant physiology is emerging. Mutation of Lin-24, an ALP derived from *Caenorhabditis elegans*, causes abnormal vulva development, leading to a failure to lay eggs^8^. Similarly, overexpression of a flower-specific ALP from the plant *Rumex acetosa* alters flower development and induces male sterility in transgenic tobacco^14^. βγ-CAT from the frog *Bombina maxima* (*B*. *maxima*) and Aep 1 from the fish *Danio rerio* have been shown to play crucial roles in antimicrobial innate immunity^15–17^. Recombinant biomphalysin, an ALP from the snail *Biomphalaria glabrata*, can directly kill parasitic *Schistosoma mansoni*, and its activity is increased in the presence of plasma, suggesting that an unknown factor could act together with biomphalysin^18^.

In our previous studies, a protein complex composed of an ALP (N-terminal βγ-crystallin domain fused with a C-terminal aerolysin domain, termed BmALP1, α subunit) and the TFF (trefoil factor domain, termed BmTFF3, β subunit), termed βγ-CAT, was identified and isolated from skin secretions of the frog *B*. *maxima*^19,20^. To our knowledge, βγ-CAT is the first example of a naturally occurring ALP and TFF complex. Importantly, previous studies revealed the capacity of βγ-CAT to modulate the cellular endolysosome pathway^15,16^. As a secreted protein complex, βγ-CAT is endocytosed via a cell membrane receptor-mediated process. After endocytosis, the oligomerization and pore formation of its BmALP1 subunit along the endolysosome pathway result in modulation of the biochemical properties of cellular endolysosomes, as observed by a change in the acidification of intracellular vesicles in the presence of the protein^15,16^. The neutralization of endocytic organelle acidification facilitated a counteraction against intracellular pathogen invasion in the frog via autophagy activation and pathogen expulsion. In addition, the BmALP1 subunit of βγ-CAT oligomerizes on endolysosomes to trigger lysosome destabilization, leading to inflammasome activation and the initiation of robust and effective antimicrobial responses^15^. However, the specific cell surface molecules mediating the actions of βγ-CAT remain elusive.

Here we discovered an interaction between βγ-CAT and acidic glycosphingolipids (AGSLs, mainly include sulfatides and gangliosides). Further detailed study revealed that the BmALP1 subunit of βγ-CAT was able to bind gangliosides, while the BmTFF3 subunit of βγ-CAT bound to sulfatides. Furthermore, both sulfatides and gangliosides in lipid rafts of the cell membrane were required for the cell binding and endocytosis of βγ-CAT. These findings revealed a double-receptor binding model of a vertebrate ALP and TFF complex, suggesting a pattern underlying the target selectivity of animal ALPs.

## Results

### βγ-CAT functions are inhibited by AGSLs

**Fig. 1:**
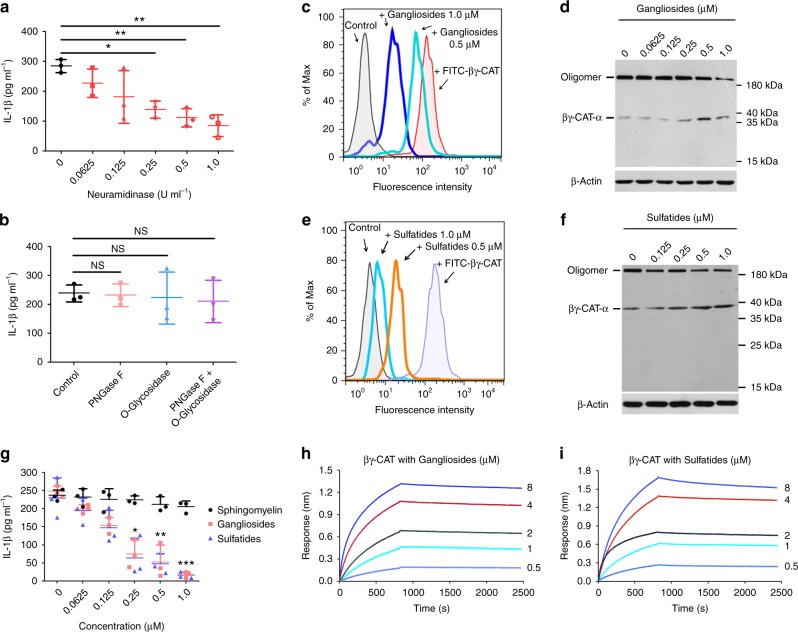
The actions of βγ-CAT were inhibited by AGSLs (gangliosides and sulfatides) via direct interactions. **a** THP-1 cells were treated with gradient concentrations of neuraminidase and then incubated with βγ-CAT. The IL-1β concentration in the supernatant was then measured by ELISA. *P < 0.05 and **P < 0.01 versus control by using unpaired two-tailed Student’s *t*-test (*n* = 3). **b** THP-1 cells were treated with different types of glycosidase and then incubated with βγ-CAT. The IL-1β concentrations in the supernatant was measured by ELISA. NS, not significant versus control by using unpaired two-tailed Student’s *t*-test (*n* = 3). **c** FITC-labeled βγ-CAT was incubated with different concentrations of gangliosides. The membrane binding of βγ-CAT to THP-1 cells was determined by flow cytometry. The untreated cells were used as a negative control. **d** βγ-CAT was incubated with different concentrations of gangliosides and added to THP-1 cells. The oligomerization of βγ-CAT was detected by western blotting. The immunoblots are representative of three independent experiments, the original images of immunoblots are shown in Supplementary Figure 7. **e** FITC-labeled βγ-CAT was incubated with different concentrations of sulfatides and then mixed with THP-1 cells. The membrane binding of βγ-CAT was determined by flow cytometry, the untreated cells were used as a negative control. **f** βγ-CAT was incubated with different concentrations of sulfatides, then mixed with THP-1 cells. The oligomerization of βγ-CAT was detected by western blotting. The immunoblots are representative of three independent experiments, the original images of immunoblots are shown in Supplementary Figure 7. **g** βγ-CAT was incubated with different concentrations of lipids and then added to LPS-primed THP-1 cells. The IL-1β concentrations in the supernatant were measured by ELISA. *P < 0.05, **P < 0.01 and ***P < 0.001 represent gangliosides and sulfatides versus sphingomyelin by two-way ANOVA with Bonferroni correction (*n* = 3). **h**, **i** The direct interaction between βγ-CAT and gangliosides (**h**) or sulfatides (**i**) was determined using a BLI assay. The BLI interaction curves are representative of three independent experiments. Bars represent the mean ± SD from three independent experiments per condition in **a**, **b** and **g**

βγ-CAT exerts its immunoregulatory activity by triggering inflammasome activation to induce IL-1β release, as we previously demonstrated^15^. Thus, an IL-1β release assay was performed to assess the functions of βγ-CAT in this study. Because no cytotoxicity was observed upon treating human acute monocytic leukemia cell line (THP-1) cells with βγ-CAT at concentrations less than 20 nM (Supplementary Fig. 1a), 5 nM βγ-CAT was used for the subsequent IL-1β release assay. First, IL-1β release induced by βγ-CAT could be inhibited by eliminating sialic acids from the THP-1 cell surface with neuraminidase (Fig. 1a), while no obvious changes were observed by treatment with other enzymes, including trypsin, chymotrypsin (Supplementary Fig. 1b), and phosphatidylinositol-specific phospholipase C (Supplementary Fig. 1c). In cell membranes, molecules containing sialic acids are mainly divided into two classes: glycoproteins and glycosphingolipids (GSLs)^21^. To identify which type of sialic acid-containing molecules were involved in the βγ-CAT actions, a glycosidase treatment assay was performed. THP-1 cells treated with PNGase F or O-glycosidase to remove the N-glycan or O-glycan of glycoproteins, respectively, showed no effects on the IL-1β release induced by βγ-CAT (Fig. 1b**)**, suggesting that the sialic acid-containing GSLs, also named gangliosides, might be involved in the actions of βγ-CAT. As one type of PFP, βγ-CAT functions depend on its membrane binding, oligomerization and pore-forming abilities. Moreover, our previous studies showed that βγ-CAT could form a sodium dodecyl sulfate -stable oligomer at approximately 180 kDa once it acted on the target cell^15,16,19^. Thus, the actions of βγ-CAT, such as membrane binding and oligomerization, were detected in this study. The membrane binding (Fig. 1c) and oligomerization (Fig. 1d) abilities of βγ-CAT in THP-1 cells were decreased by incubation with gangliosides. In addition to gangliosides, sulfatides are another predominant type of sulfate substituent containing AGSLs in the cell membranes of vertebrates and are also primarily found at the plasma membrane outer leaflet of most eukaryotic cells^22^. In agreement with the inhibitory effect of gangliosides, the membrane binding (Fig. 1e) and oligomerization abilities (Fig. 1f) of βγ-CAT were largely attenuated by incubation with sulfatides. Furthermore, the IL-1β release of THP-1 cells induced by βγ-CAT was largely attenuated by incubation with gangliosides or sulfatides in a dose-dependent manner but was not affected by incubation with sphingomyelin, a type of sphingolipid that shares a structure similar to those of gangliosides and sulfatides but has no glycan headgroups (Fig. 1g). In addition, no inhibitory effects were observed by incubation with other sphingolipids sharing a common sphingosine backbone with AGSLs, such as sphingosine, ceramide and cerebroside (Supplementary Fig. 1d). Furthermore, direct interactions between βγ-CAT and gangliosides (Fig. 1h) or sulfatides (Fig. 1i) in vitro were observed based on the biolayer interferometry (BLI) assay. The K_D_ values of βγ-CAT with gangliosides and sulfatides were (5.67 ± 0.23) × 10^−8^ M and (6.55 ± 0.15) × 10^−8^ M, respectively. These findings suggest that AGSLs (gangliosides or sulfatides) rather than other sphingolipids inhibit the actions of βγ-CAT via direct interactions.

As described above, the actions of βγ-CAT were inhibited by AGSLs; therefore, we evaluated whether the functions of βγ-CAT were also impacted by other acidic molecules, revealing that IL-1β release induced by βγ-CAT was not affected by incubation with heparin until concentrations reaching 100 μM (Supplementary Fig. 1e). These results indicate that AGSLs can specifically interact with βγ-CAT and inhibit its actions.

AGSLs of the cell membrane exist as different structural subtypes, such as GM1, GM3, GD1a and GT1b^23^. In a subsequent study, we examined the inhibitory activities of eight types of gangliosides (GM1, GM2, GM3, GM4, GD1a, GD1b, GD3 and GT1b) on βγ-CAT, and no differences in the inhibition of IL-1β release induced by βγ-CAT were observed among these types of gangliosides (Supplementary Fig. 1f). Furthermore, there were no differences in the binding between βγ-CAT and most of the gangliosides except GD1b and GT1b, which had relatively weak binding (Supplementary Fig. 2 and Supplementary Table 1). These findings suggest that βγ-CAT targets AGSLs rather than one type of AGSL. Taken together, our findings show that AGSLs can interact with βγ-CAT directly and thereby inhibit its actions and activities.

### βγ-CAT domain binding specificity to AGSLs

Typical AGSLs are composed of a common ceramide backbone and an acidic glycan head. To identify the part of the AGSLs involved in the interaction with βγ-CAT, four sphingolipids sharing a structural correlation with AGSLs were used for the binding assay: sphingosine (the basic skeleton of AGSLs), ceramide (sphingolipids without glycan), sphingomyelin (the glycan of AGSLs is replaced with phosphatidylcholine or phosphatidylethanolamine) and cerebroside (neutral GSL). The BLI assay showed no direct interactions between βγ-CAT and any of the four sphingolipids excluding AGSLs (gangliosides or sulfatides) (Supplementary Fig. 3). These results suggest that βγ-CAT binds to the extracellular acidic glycan headgroup rather than the ceramide backbone of AGSLs.

**Fig. 2:**
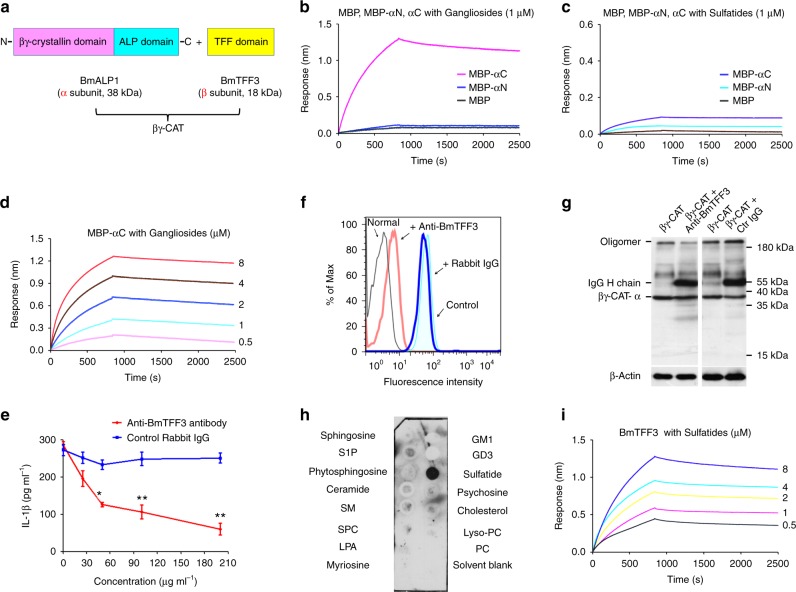
The BmALP1 and BmTFF3 subunits of βγ-CAT bound to gangliosides and sulfatides, respectively. **a** Schematic graph of βγ-CAT, BmALP1 subunit and BmTFF3 subunit. The BmALP1 subunit and BmTFF3 subunit of βγ-CAT have apparent molecular weights of 38 kDa and 18 kDa, respectively. **b** Interactions between MBP-αN and MBP-αC with gangliosides was detected by BLI assay. **c** Interactions between MBP-αN and MBP-αC with sulfatides was detected by BLI assay. **d** The binding kinetic curves between MBP-αC and gangliosides were determined by BLI assay. **e** βγ-CAT was incubated with different concentrations of anti-BmTFF3 polyclonal antibody or control IgG and added to LPS-primed THP-1 cells. The IL-1β concentration in the supernatant was measured by ELISA. Bars represent the mean ± SD from three independent experiments. *P < 0.05 and **P < 0.01 vs. control IgG by using unpaired two-tailed Student’s *t* test (*n* = 3). **f** FITC-labeled βγ-CAT was incubated with an anti-BmTFF3 polyclonal antibody or control rabbit IgG and added to THP-1 cells, the binding of βγ-CAT with THP-1 cells was evaluated by flow cytometry. Untreated THP-1 cells are indicated as normal, and THP-1 cells treated with FITC-labeled βγ-CAT are indicated as the control. **g** βγ-CAT was incubated with an anti-BmTFF3 polyclonal antibody or control rabbit IgG and then added to THP-1 cells, the oligomerization of βγ-CAT was detected by western blotting. The immunoblots are representative of three independent experiments, the original images of immunoblots are shown in Supplementary Figure 8. **h** The binding of BmTFF3 with different types of sphingolipids was determined by protein-lipid overlay assay. The blots are representative of three independent experiments, the original images of blots are shown in Supplementary Figure 8. **i** The binding kinetic curves between BmTFF3 and sulfatides was determined by BLI assay. The BLI interaction curves in **b**–**d** and **i** are representative of three independent experiments

As described in the introduction, βγ-CAT is composed of three domains: a βγ-crystallin domain, an ALP domain and a TFF domain (Fig. 2a). We next investigated which βγ-CAT domain is involved in its interaction with AGSLs. The recombinant maltose binding protein-fused βγ-crystallin domain (MBP-αN) and maltose binding protein-fused ALP domain (MBP-αC) were examined in a binding assay. Gangliosides showed a stronger interaction with MBP-αC than with MBP-αN or MBP in the BLI assay (Fig. 2b). Surprisingly, neither MBP-αN nor MBP-αC had a ability to bind sulfatides in the BLI assay (Fig. 2c). Detailed binding kinetics curve analyses were performed, and the calculated *K*_D_ value of MBP-αC with gangliosides was approximately (8.06 ± 0.33) × 10^−8^ M (Fig. 2d). These findings suggest that the ALP domain of βγ-CAT rather than the βγ-crystallin domain specifically binds to gangliosides but not sulfatides. In addition, neither the ALP domain nor the βγ-crystallin domain of βγ-CAT is involved in its binding with sulfatides. In contrast to typical ALPs, βγ-CAT was the first identified secreted ALP and TFF complex, therefore, the roles of its BmTFF3 subunit in the complex needed to be elucidated. First, the IL-1β release in THP-1 cells induced by βγ-CAT was largely attenuated by incubation with anti-BmTFF3 polyclonal antibodies but not with normal rabbit IgG (Fig. 2e). Furthermore, the membrane binding (Fig. 2f) and oligomerization (Fig. 2g) abilities of βγ-CAT were largely attenuated by incubation with anti-BmTFF3 polyclonal antibodies at a concentration of 100 μg ml^−1^, suggesting that the BmTFF3 subunit is essential for the actions of βγ-CAT. The BmTFF3 subunit of βγ-CAT is difficult to express as a recombinant protein because it contains 9 disulfide bonds. Fortunately, natural BmTFF3 purified from frog skin secretions was used in the subsequent study (Supplementary Fig. 4). The following protein-lipid overlay assay revealed that BmTFF3 showed a high binding to sulfatides but no obvious binding to other sphingolipids, including gangliosides (Fig. 2h). Moreover, a direct interaction between BmTFF3 and sulfatides was observed using the BLI assay, and the calculated K_D_ value was approximately (6.23 ± 0.27) × 10^−8^ M (Fig. 2i). Taken together, these findings suggest that βγ-CAT binds to the glycan headgroups of AGSLs rather than to the ceramide backbone. Furthermore, the BmALP1 subunit of βγ-CAT binds to gangliosides, while the BmTFF3 subunit of βγ-CAT binds to sulfatides.

### Membrane lipid rafts mediate the actions **o**f βγ-CAT

Lipid rafts are microdomains on the plasma membrane enriched in cholesterol, sphingolipids and glycosylphosphatidylinositol (GPI)-anchored proteins that play vital roles in many cellular processes, such as signal transduction, membrane trafficking, and pathogen entry^24^. Most AGSLs are known to exist in membrane lipid raft microdomains^25^. Thus, we next investigated whether membrane lipid rafts mediate the actions of βγ-CAT.

**Fig. 3:**
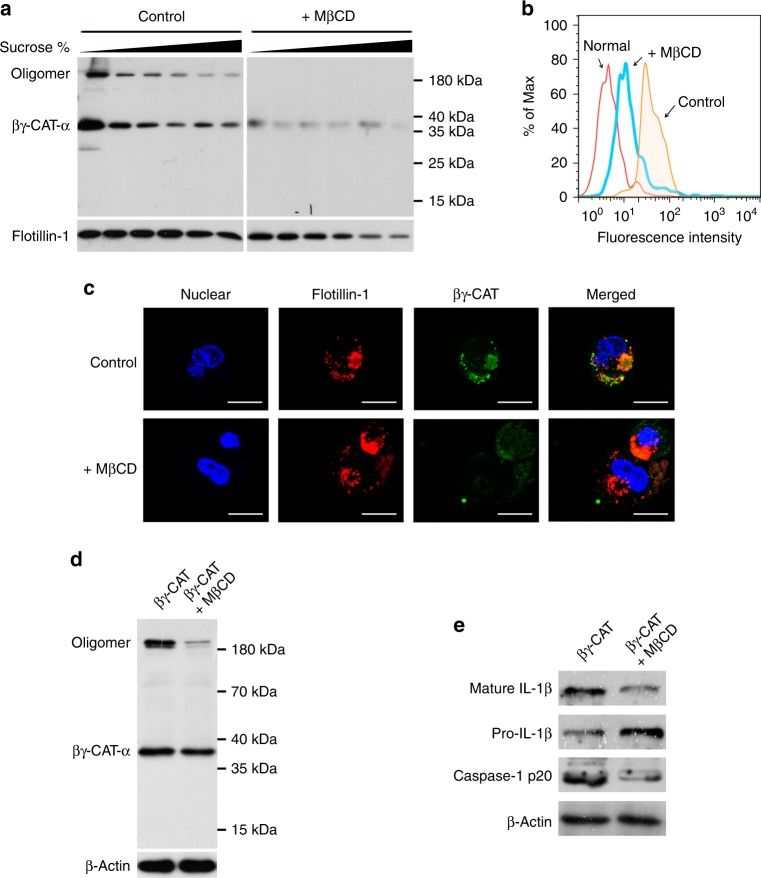
Membrane lipid rafts mediated the binding, endocytosis, oligomerization and activity of βγ-CAT. **a** Frog peritoneal cells were treated with MβCD and then incubated with βγ-CAT. The detergent-resistant membrane fractions were extracted by density gradient centrifugation, and the distribution of βγ-CAT on membrane lipid rafts was determined by western blotting. Flotillin-1 is a marker of lipid rafts. The immunoblots are representative of three independent experiments, the original images of immunoblots are shown in Supplementary Figure 9. **b** Frog peritoneal cells were treated with MβCD and then incubated with FITC-labeled βγ-CAT. The binding of βγ-CAT to peritoneal cells was evaluated by flow cytometry. The untreated normal frog peritoneal cells are indicated as normal, and frog peritoneal cells treated with FITC-labeled βγ-CAT are indicated as control. **c** The colocalization of βγ-CAT and membrane lipid rafts was observed by confocal microscopy. Scale bar = 10 μm. **d** Frog peritoneal cells were treated with MβCD and then incubated with βγ-CAT. The oligomerization of βγ-CAT was detected by western blotting. The immunoblots are representative of three independent experiments, the original images of immunoblots are shown in Supplementary Figure 10. **e** The caspase-1 activation and IL-1β release induced by βγ-CAT was measured by western blotting. The immunoblots are representative of three independent experiments, the original images of immunoblots are shown in Supplementary Figure 11

To identify the location of βγ-CAT at the membrane, detergent-resistant membranes^26^, also defined as lipid raft fractions, were extracted from frog peritoneal cells after treatment with 100 nM βγ-CAT for 30 min. Western blot analysis revealed that βγ-CAT was mainly enriched in the lipid raft fractions of frog peritoneal cells; however, enrichment of βγ-CAT in lipid rafts was attenuated after lipid raft disruption by the addition of 5 mM methyl-β-cyclodextrin (MβCD) to remove cholesterol (Fig. 3a). In addition, flow cytometry analysis showed that the membrane binding between frog peritoneal cells and βγ-CAT was inhibited by treatment with MβCD under the same conditions (Fig. 3b). Confocal microscopy observations showed that the colocalization between βγ-CAT and flotillin-1, a marker of lipid rafts, was largely attenuated after peritoneal cell treatment with MβCD (Fig. 3c). Further study showed that the oligomerization ability of βγ-CAT was almost completely inhibited by treatment with MβCD (Fig. 3d). Finally, both caspase-1 activation and the release of mature IL-1β induced by βγ-CAT were attenuated in frog peritoneal cells after treatment with MβCD (Fig. 3e). These findings suggest that the membrane lipid raft microdomains act as anchor sites and mediate the actions of βγ-CAT, including membrane binding, endocytosis, oligomerization and pore formation.

### AGSLs in lipid rafts are essential for the actions of βγ-CAT

**Fig. 4:**
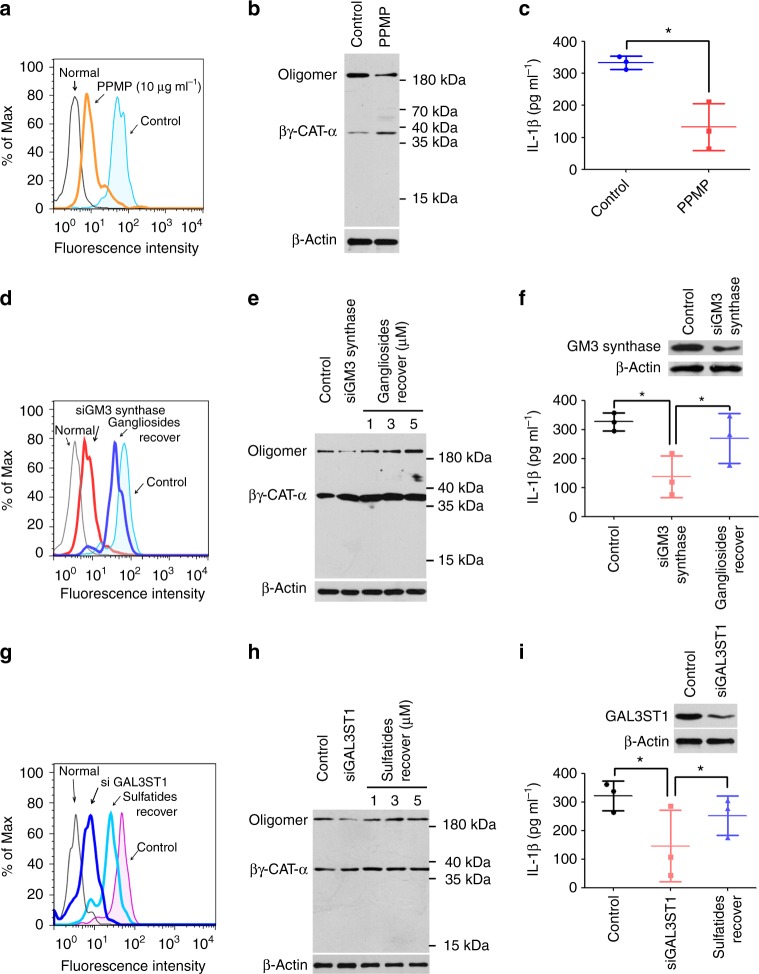
AGSLs of the cell membrane were essential for the actions of βγ-CAT. **a** THP-1 cells were treated with PPMP and then incubated with FITC-labeled βγ-CAT. The binding between βγ-CAT and THP-1 cells was detected by flow cytometry. **b** The oligomerization of βγ-CAT was determined by western blotting. The immunoblots are representative of three independent experiments, the original images of immunoblots are shown in Supplementary Figure 12. **c** The IL-1β release induced by βγ-CAT was measured by ELISA. *P *<* 0.05 vs. control by using unpaired two-tailed Student’s *t* test (*n* = 3). **d**–**f** THP-1 cells in which ganglioside expression was knocked down or gangliosides re-addition, then incubated with βγ-CAT. The binding between βγ-CAT and THP-1 cells was detected by flow cytometry (**d**), the oligomerization of βγ-CAT was detected by western blotting (**e**), and the IL-1β concentration was measured by ELISA (**f**). *P *<* 0.05 vs. the respective control by using unpaired two-tailed Student’s *t* test (*n* = 3). The immunoblots (**e**) are representative of three independent experiments, the original images of immunoblots are shown in Supplementary Figure 13. **g**–**i** THP-1 cells in which sulfatide expression was knocked down or sulfatides re-addition, then incubated with βγ-CAT. The binding between βγ-CAT and THP-1 cells was detected by flow cytometry (**g**), the oligomerization of βγ-CAT was detected by western blotting (**h**), and the IL-1β concentration was measured by ELISA (**i**). *P *<* 0.05 vs. the respective control by using unpaired two-tailed Student’s *t* test (*n* = 3). The immunoblots (**h**) are representative of three independent experiments, the original images of immunoblots are shown in Supplementary Figure 13. In flow cytometry (**a**, **d**, **g**), the untreated THP-1 cells are indicated as normal, and THP-1 cells treated with FITC-labeled βγ-CAT are indicated as control. Bars represent the mean ± SD from three independent experiments per condition in **c**, **f**, **i**

As mentioned above, almost all AGSLs are exclusively distributed in membrane lipid rafts, and these raft microdomains have been shown to mediate the actions of βγ-CAT. Thus, we assessed whether the AGSLs of membrane rafts were essential for the actions of βγ-CAT. To study the effects of membrane AGSLs on the actions of βγ-CAT, human THP-1 cells were subjected to subsequent RNAi-mediated knockdown or pharmacological inhibition of synthetic GSL enzymes. The biosynthesis of GSLs can be substantially inhibited by 1-phenyl-2-palmitoylamino-3-morpholino-1-propanol (PPMP), a specific pharmacological inhibitor of glucosylceramide synthase^27^. Flow cytometry analysis showed that the expression of gangliosides on the surface of THP-1 cells was decreased by treatment with 10 μg ml^−1^ PPMP at 37 °C for 28 h (Supplementary Fig. 5a). The actions of βγ-CAT on THP-1 cells, including its membrane binding (Fig. 4a), oligomerization (Fig. 4b) and IL-1β release abilities (Fig. 4c), were largely attenuated by inhibiting the expression of AGSLs in THP-1 cells with 10 μg ml^−1^ PPMP. For ganglioside biosynthesis, GM3 serves as a common precursor, and GM3 synthase is the key enzyme in ganglioside biosynthesis^28^. Therefore, a GM3 synthase shRNA lentivirus was used to knockdown the expression of gangliosides on the cell surface (Supplementary Fig. 5b). The membrane binding (Fig. 4d), oligomerization (Fig. 4e) and IL-1β release (Fig. 4f) induced by βγ-CAT in THP-1 cells were inhibited after knockdown of GM3 synthase. Interestingly, the actions of βγ-CAT were recovered after the readdition of free gangliosides to the GM3 synthase knockdown THP-1 cells (Fig. 4d–f). These findings show that cell surface gangliosides are essential for the actions of βγ-CAT. Similarly, to test the requirement of sulfatides for βγ-CAT actions, cerebroside sulfotransferase (CST, also termed GAL3ST1), a key enzyme in sulfatide biosynthesis, was knocked down using GAL3ST1 shRNA lentiviral particles. Consistent with the knockdown of GM3 synthase, the expression of sulfatides in the membrane was decreased after GAL3ST1 was knocked down, and the membrane sulfatides recovered when free sulfatides were added back (Supplementary Fig. 5c). Furthermore, the membrane binding (Fig. 4g), oligomerization (Fig. 4h), and IL-1β release (Fig. 4i) induced by βγ-CAT were largely attenuated after sulfatide expression was knocked down. Interestingly, all of the actions of βγ-CAT were recovered following the readdition of free sulfatides to the GAL3ST1 knockdown THP-1 cells (Fig. 4g–i). These findings suggest that the two types of AGSLs on cell membranes (gangliosides and sulfatides) are equally important for the actions of βγ-CAT. Taken together, our findings indicate that both gangliosides and sulfatides of membrane lipid rafts are essential for the actions of βγ-CAT.

### Frog AGSLs mediate the immune response triggered by βγ-CAT

**Fig. 5:**
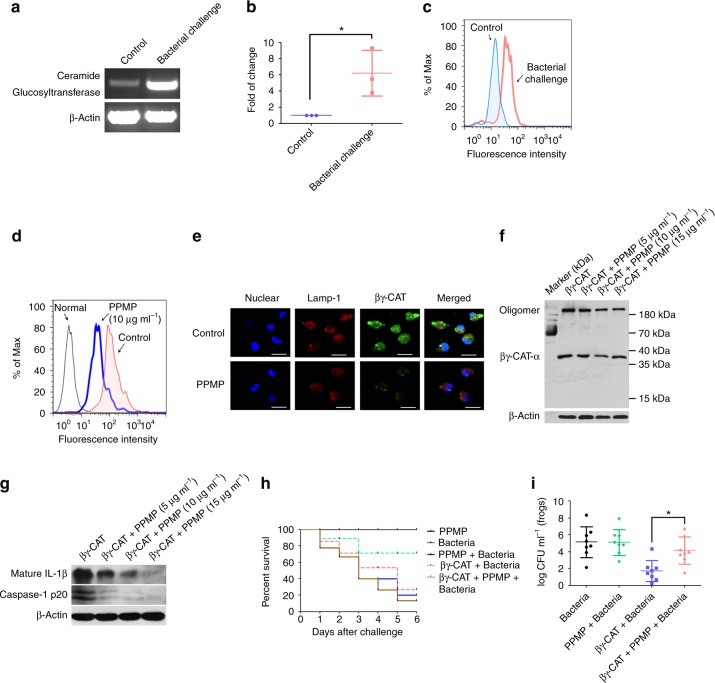
AGSLs mediate the antimicrobial innate immune response triggered by βγ-CAT. **a**, **b** Bacteria-challenged peritoneal cells were collected, and the expression levels of ceramide glucosyltransferase in peritoneal cells were determined by PCR (**a**) and RT-qPCR (**b**) using specific primers. Bars represent the mean ± SD from three independent experiments. *P *<* 0.05 versus control by using unpaired two-tailed Student’s *t* test (*n* = 3). The gels (**a**) are representative of three independent experiments, the original full-gel images are shown in Supplementary Figure 14. **c** The expression levels of gangliosides in the peritoneal cell membrane were detected by flow cytometry. Unchallenged frog peritoneal cell served as the negative control. **d** Peritoneal cells were treated with PPMP and then incubated with FITC-labeled βγ-CAT. The binding between βγ-CAT and peritoneal cells was detected by flow cytometry. Untreated normal frog peritoneal cells are indicated as normal, frog peritoneal cells treated with FITC-labeled βγ-CAT are indicated as control. **e** The endocytosis and lysosomal colocalization of βγ-CAT were observed by confocal microscopy. Scale bar = 25 μm. **f** Peritoneal cells were treated with PPMP and then incubated with βγ-CAT. The oligomerization of βγ-CAT was detected by western blotting. The immunoblots are representative of three independent experiments, the original images of immunoblots are shown in Supplementary Figure 15. **g** LPS-primed peritoneal cells were treated with different concentrations of PPMP and then incubated with βγ-CAT. Caspase-1 activation and mature IL-1β release induced by βγ-CAT were determined by western blotting. The immunoblots are representative of three independent experiments, the original images of immunoblots are shown in Supplementary Figure 16. **h**, **i** Frogs (*B*. *maxima*) were intraperitoneally injected with PPMP before the injection of βγ-CAT and bacteria (*A*. *hydrophila*). Frogs mortality (**h**) was then observed daily (*n* = 8). The number of peritoneal bacteria (**i**) was then counted 48 h after infection. Bars represent the mean ± SD, *P *<* 0.05 vs. the respective control by using unpaired two-tailed Student’s *t* test (*n* = 8). The survival rate and bacterial count data are representative of two experiments

A previous study showed that βγ-CAT can protect frogs against pathogen invasion by triggering the rapid innate immune response mediated by inflammasome activation and the subsequent IL-1β release^15^. We next investigated whether AGSLs mediated the βγ-CAT-induced antimicrobial innate immunity response, and βγ-CAT at concentrations reaching 400 nM exerted no cytotoxic effects on frog peritoneal cells (Supplementary Fig. 6a); thus, 100 nM βγ-CAT was used for the subsequent assays. Because no specific pharmacological inhibitor was used to block sulfatide synthesis, only the anti-GM3 antibody was used to detect gangliosides in frogs. In *B*. *maxima*, ceramide glucosyltransferase, the key enzyme catalyzing ganglioside synthesis, was confirmed by BLAST by screening the human enzyme against the whole *B*. *maxima* transcriptome using previously described methods^29^. The analysis in mRNA levels showed that the expression of frog ceramide glucosyltransferase were upregulated after 6 h of exposure to *Aeromonas hydrophila* (*A*. *hydrophila*) (Fig. 5a, b). Flow cytometry analysis revealed that the expression of gangliosides in frog peritoneal cells was increased after challenge with *A*. *hydrophila* (Fig. 5c). To further investigate the importance of frog gangliosides in the βγ-CAT-triggered innate immunity response, a blockade of ganglioside biosynthesis was performed using PPMP as described above. PPMP at concentrations up to 25 μg ml^−1^ exerted no cytotoxic effects on frog peritoneal cells (Supplementary Fig. 6b). Thus, 10 μg ml^−1^ PPMP was used in the subsequent pharmacological inhibitor blockade assay. First, the gangliosides on the cell surface of frog peritoneal cells were largely decreased after treatment with 10 μg ml^−1^ PPMP (Supplementary Fig. 6c). Furthermore, not only the membrane binding (Fig. 5d), endocytosis (Fig. 5e) and oligomerization abilities (Fig. 5f) of βγ-CAT decreased, but the caspase-1 activation and mature IL-1β release induced by βγ-CAT were also largely attenuated after the gangliosides of frog peritoneal cells were eliminated with PPMP (Fig. 5g). These findings suggest that the gangliosides of frogs mediate the inflammasome-associated events induced by βγ-CAT. To further examine the roles of gangliosides in the microbial clearance of frogs, the frog peritoneal bacterial infection model was used. The abilities of βγ-CAT to prolong the survival rate of infected frogs (Fig. 5h) and induce rapid bacterial clearance (Fig. 5i) were largely attenuated by intraperitoneal injection with 100 μg kg^−1^ PPMP at 36 h before intraperitoneal injection with βγ-CAT or the following bacteria, while PPMP alone had no impact on survival or bacterial clearance in frogs. Taken together, these findings show that the AGSLs of frogs mediate the antimicrobial innate immunity response triggered by βγ-CAT.

## Discussion

The major challenges to investigate the numerous ALPs in animals and plants are illustrating their natural molecule compositions, biological functions and acting patterns or pathways. βγ-CAT is an ALP and TFF complex that was identified in the frog *B*. *maxima* and represents the first example of an endogenous secretive ALP targeting and regulating the cellular endolysosome pathway^15,16,19^. Previous studies have illustrated that the acting pathway of βγ-CAT is characterized by the receptor-mediated endocytosis of its BmALP1 subunit. The subsequent oligomerization and pore formation of BmALP1 along the cellular endolysosome pathway results in the modulation of intracellular vesicles, which could lead to diverse cellular responses and outcomes, such as unconventional secretion as well as pathogen elimination^15,16^. Cell surface molecules that mediate the binding and endocytosis of βγ-CAT are clearly key elements in the initiation of its cellular effects. In the present study, we observed an interaction between βγ-CAT and AGSLs, and cell surface AGSLs (gangliosides and sulfatides) in lipid rafts are key elements for the binding and endocytosis of βγ-CAT, both of which are required. Furthermore, each βγ-CAT subunit binds to one type of AGSL.

AGSLs are defined as a type of GSL that contains one or more acidic sugar residues. In vertebrates, mainly two types of AGSLs exist, gangliosides containing sialic acids and sulfatides containing sulfate substituents^30^, with gangliosides being specific membrane elements that are found mainly in vertebrates. These two types of AGSLs have not been found in prokaryotic cells, such as bacteria^22,31,32^. The results presented herein are consistent with our previous findings that βγ-CAT acts as an endogenous effective molecular complex and targets the cells of the frog *B*. *maxima* to exert its biological effects but has no direct killing effect on bacteria^15^. As a secreted protein complex, βγ-CAT must be endocytosed to exert its intracellular activity^16^. Consistently, AGSLs are primarily localized in the outer leaflet of the plasma membrane, where the long saturated hydrocarbon chains of ceramide anchor to lipid rafts of the plasma membrane and glycan extends into the extracellular space^33^. βγ-CAT directly interacts with the extracellular glycan headgroup of AGSLs rather than the ceramide region embedded in the lipid bilayer (Supplementary Fig. 3). Consistent with this finding, it is worth noting that AGSLs are targets of many pathogens for endocytosis and intracellular invasion^34^. Finally, AGSLs are involved in many vital physiological and pathological functions, including immunity^35,36^. The present study indicated that the expression of gangliosides in host immune cells was upregulated during bacterial challenge, and the elimination of gangliosides in immune cells led to an impaired innate immune response and microbial clearance stimulated by βγ-CAT (Fig. 5). These results not only emphasized the role of gangliosides in innate immunity but also revealed a link between gangliosides and ALP in vertebrate immune responses.

Most AGSLs are located in the lipid raft microdomains of membranes. Lipid rafts are small (10–200 nm), heterogeneous, and highly dynamic cholesterol- and sphingolipid-enriched domains that compartmentalize cellular processes^37^. It has been well documented that lipid rafts mediate many endocytosis processes, including the internalization of ligands and receptors^38^ as well as the entry of pathogens^39^. MβCD functions as a lipid raft disruption agent by removing cholesterol, and the cholesterol depletion induces the disassembly of lipid rafts and leads to the diffusion of AGSLs distributed in rafts. In the present study, the actions of βγ-CAT were largely attenuated after treatment with MβCD (Fig. 3), indicating that lipid rafts are the key membrane microdomains necessary for anchoring the actions of βγ-CAT, as they affect the concentration of AGSLs necessary for its binding and endocytosis abilities. Lipid rafts are highly dynamic microdomains, and various types of lipid rafts are found in cell membranes with distinct lipid and protein compositions^40^. Whether βγ-CAT selectively targets a specific type of lipid raft (containing at least both gangliosides and sulfatides at a proper ratio) and its molecular composition and unique biophysical properties are interesting future challenges.

The present study revealed that the BmALP1 subunit of βγ-CAT bound to gangliosides, while the BmTFF3 subunit of βγ-CAT bound to sulfatides (Fig. 2). Interestingly, each βγ-CAT subunit recognized different extracellular glycan headgroups of AGSLs. In fact, the binding between ALP and sugars has been previously reported. Buckley et al. found that a GPI-anchored glycoprotein on the plasma membrane was the receptor for aerolysin from *A*. *hydrophila*^41^. The lectin-like domain of aerolysin is responsible for binding to modified N-linked sugars and the GPI anchor glycan core^42^. Dln1, a jacalin domain-fused ALP from zebrafish, is capable of binding to high-mannose glycans^43^. In contrast to these ALPs, the BmALP1 subunit of βγ-CAT specifically bound to the sialic acid-containing glycan of gangliosides, and this was the first example of an interaction between a vertebrate-derived ALP and gangliosides. As described previously, the BmALP1 subunit of βγ-CAT comprises a βγ-crystallin domain-fused ALP^15^. However, the βγ-crystallin domain of BmALP1 subunit did not display binding to gangliosides (Fig. 2b), suggesting that the βγ-crystallin domain of βγ-CAT may play other important roles, such as stabilizing the complex structure or acting as a regulatory domain in the action of the protein, which will be an interesting topic to address in future studies. Human TFF2 is a lectin that binds to α-GlcNAc-capped mucin glycans^44^. We reported herein that the BmTFF3 subunit of βγ-CAT showed a high affinity for sulfatides, while it had no binding activity with other sphingolipids without sulfonated galactose (Fig. 2h). On the other hand, while the functions of βγ-CAT were not impacted by the treatment of cells with different proteases (Supplementary Fig. 1b, 1c), it is possible that unknown membrane proteins could act as coreceptors and thus affect the actions of βγ-CAT. Taken together, our findings suggest that this vertebrate-derived ALP and TFF complex exhibits a double binding model that is distinct from those of single-chain ALPs, such as aerolysin or lysenin, which act on one specific membrane molecule, such as GPI-anchored glycoproteins and sphingomyelin^41,45^. Double binding obviously provides a reasonable explanation for the selectivity and specificity of βγ-CAT.

It is worth noting that, in the present study, the free BmTFF3 subunit of βγ-CAT could be readily purified from *B*. *maxima* skin secretions (Supplementary Fig. 4). BmTFF3 has a signal peptide in its precursor^19^, and it should be secreted extracellularly after synthesis via the classical secretory pathway. In contrast, the BmALP1 subunit of βγ-CAT lacks a signal peptide in its precursor and might be released via an unconventional secretory pathway. This finding suggested the presence of an assembly process to form the natural active βγ-CAT complex under specific conditions, which should be tightly regulated. The interaction between BmALP1 and BmTFF3 during the assembly of this executive protein complex and its regulatory mechanisms are certainly important questions that should be addressed in future studies. On the other hand, it is worth of pointing out that recent studies has also showed that βγ-CAT is able to promote wound healing and tissue repair^10,19^, indicating that the protein complex could stimulate cell migration, proliferation and differentiation directly and/or indirectly. Further investigation certainly will uncover the detailed mechanisms inside related to βγ-CAT endocytosis and its regulation on endolysosome system.

**Fig. 6:**
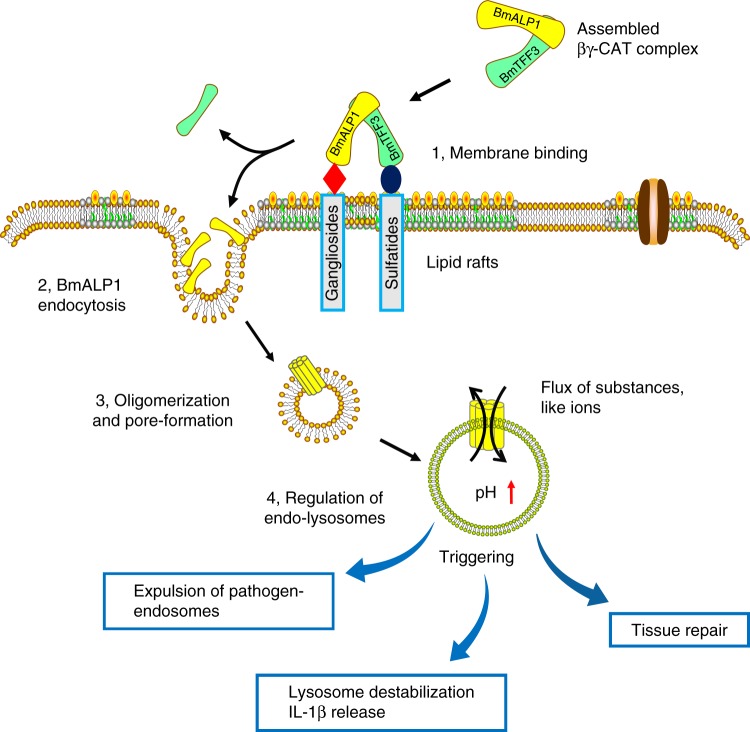
The proposed action model and pathway of βγ-CAT. As a vertebrate-secreted ALP and TFF protein complex, βγ-CAT exerts its functions via an assembly process. The actions of assembled βγ-CAT can be divided into four steps. Step 1. Membrane binding. Once the assembled βγ-CAT encounters target cells, βγ-CAT is subsequently anchored to lipid raft microdomains on the plasma membrane. Therefore, the BmALP1 subunit of βγ-CAT binds to the extracellular glycan headgroup of gangliosides, and the BmTFF3 subunit of βγ-CAT binds to the extracellular glycan headgroup of sulfatides. Step 2. After βγ-CAT binding to the membrane, the BmTFF3 subunit detaches from the membrane, and the BmALP1 subunit of βγ-CAT is endocytosed and enters the cell. Step 3. The endocytic βγ-CAT then oligomerizes and forms pores in the membranes of endolysosomes. Step 4. The formed pores induce a flux of substances, such as ions, ultimately regulating the properties of endolysosomes. As we observed previously, changes in the properties of endolysosomes can trigger various effects, such as the expulsion of pathogen-containing vesicles^16^ and increased lysosome destabilization, leading to inflammasome-dependent IL-1β release^15^ and tissue repair^10^

In conclusion, we herein elucidated that an endogenous secreted pore-forming protein complex, βγ-CAT, targeted AGSLs in lipid rafts to initiate its endocytosis and subsequent regulation of endolysosomes, ultimately triggering a series of physiological reactions, such as lysosome destabilization, IL-1β release, pathogen-endosome expulsion and tissue repair (Fig. 6**)**. Both gangliosides and sulfatides are required for the actions of βγ-CAT, as its ALP subunit binds to the former, and its TFF subunit binds to the latter. These findings revealed a previously unknown double binding pattern of a secreted ALP and TFF complex in animals, representing an acting pattern for the target selectivity of animal ALPs.

## Methods

### Animals

The collection and feeding of frogs (*B*. *maxima*) were performed as described previously^16^. All procedures and the care and handling of animals were approved by the Ethics Committee of the Kunming Institute of Zoology, the Chinese Academy of Sciences.

### Cell lines, antibodies and reagents

THP-1 cell was purchased from the American Type Culture Collection (Manassas, VA, USA) and maintained in growth medium as recommended by the ATCC. Mixed gangliosides, monosialoganglioside, GM1, GM2, GM3, disialoganglioside, GD1a, GD1b, GD3, sphingosine, ceramides, cerebrosides, trisialoganglioside GT1b and glucosylceramide synthase inhibitor PPMP were purchased from Matreya LLC (State College, PA, USA). Asialoganglioside-GM1 and monosialoganglioside GM4 were purchased from Merck Millipore (Darmstadt, Germany). Sulfatides were purchased from Avanti Polar Lipids, Inc. (Alabaster, Alabama, USA). Sialic acid (N-Acetyl neuraminic acid), sphingomyelin, trypsin, chymotrypsin, phosphatidylinositol-specific phospholipase C, sulfatase, MβCD, and mouse anti-O4 monoclonal antibody (O7139) were purchased from Sigma-Aldrich (St. Louis, MO, USA). The neuraminidase was purchased from Roche (Mannheim, Baden-Württemberg, Germany). The PNGase F and *O*-glycosidase were purchased from New England Biolabs, Inc (Ipswich, MA, USA). The anti-monosialoganglioside GM3 monoclonal antibody (M2590) was purchased from Cosmo Bio Co, LTD (Tokyo, Japan). The GM3 synthase antibody (B-12), GM3 synthase shRNA (h) lentiviral particles (sc-72297-V), GAL3ST1 shRNA (h) lentiviral particles (sc-75081-V), GAL3ST1 antibody (sc-86462), control shRNA lentiviral particles-A (sc-108080), polybrene (sc-134220) and puromycin dihydrochloride (sc-108071) were purchased from Santa Cruz Biotechnology (Santa Cruz, CA, USA). The Alexa Fluor 488-conjugated goat anti-mouse IgG/IgM (H + L) secondary antibody and lactate dehydrogenase (LDH) Cytotoxicity Assay Kit were purchased from Thermo Fisher Scientific Inc. (Waltham, MA, USA). The Amine Reactive Second Generation (AR2G) Tray (18-5092) was purchased from Pall FortéBio, LLC (Menlo Park, CA, USA), Sphingo strips (S-6000) was purchased from Echelon Biosciences Inc. (Salt Lake City, UT, USA). An ELISA kit for detecting human IL-1β was purchased from MultiSciences (Hangzhou, Zhejiang, China). The HRP-conjugated goat anti-rabbit IgG (H + L) secondary antibody (BA1054) was purchased from BOSTER (Wuhan, Hubei, China).

### Measurements of IL-1β levels and enzymatic treatment assays

IL-1β levels were measured according to a previously described procedure^46^. In brief, adherent THP-1 cells were induced by 100 ng ml^−1^ phorbol 12-myristate 13-acetate (PMA), and the medium containing PMA was subsequently removed and replaced with complete DMEM/F12 medium containing 10% fetal bovine serum (FBS). For detection of IL-1β, THP-1 cells were first primed with lipopolysaccharide (LPS) at a concentration of 100 ng ml^−1^ for 2 h at 37 °C and then incubated with various concentrations of βγ-CAT or a βγ-CAT/lipids mixture for 2 h at 37 °C. Then, the culture supernatants were collected by centrifugation at 1000 × *g* for 10 min, and the concentration of IL-1β was determined using a commercial ELISA kit (MultiSciences) according to the manufacturer’s instructions.

For the enzyme treatment assay of THP-1 cells, adherent THP-1 cells were washed three times with cold phosphate buffered saline (PBS) and then mixed with different concentrations of trypsin, chymotrypsin (0.5–2.5 mg ml^−1^), neuraminidase (0–1.0 U ml^−1^) or phosphatidylinositol-specific phospholipase C (0–2.5 U ml^−1^) and incubated for 2 h at 37 °C. Then, the supernatant was discarded, and the remaining pellet was washed three times with cold PBS. The washed THP-1 cells were then used for the subsequent IL-1β release assay. THP-1 cells were incubated with PBS as a negative control.

### The inhibition of βγ-CAT functions by lipids in vitro

To study the inhibitory activities of various lipids on βγ-CAT in vitro, an in vitro incubation assay was performed. Briefly, βγ-CAT at 5 nM was mixed with different concentrations of AGSL or sphingomyelin (0, 0.0625, 0.125, 0.25, 0.5 and 1 μM) and incubated at 37 °C in a water bath for 30 min. Subsequently, the βγ-CAT and sphingolipids mixture was added to LPS-primed THP-1 cells, and the mixture was incubated for 2 h at 37 °C. Next, the culture supernatants were collected to detect the concentration of IL-1β, and the cells were collected for the detection of βγ-CAT oligomerization.

### LDH release detection

For the LDH release assay, THP-1 cells or frog peritoneal cells were cultured on a 96-well plate. Next, the complete medium was removed from the wells and replaced with FBS-free medium, and the cells were incubated for 2 h. Subsequently, the medium of each well was removed, and different concentrations of βγ-CAT were added. After incubation for 2 h, the cell supernatants were collected, and LDH release was detected according to the manufacturer’s instructions. Cells treated with 0.1% Triton X-100 were used to define 100% LDH release, and cells treated with FBS-free medium served as a negative control.

### Flow cytometry assay

The flow cytometry methods used in the present study were similar to those mentioned in our previous report^47^. To detect the expression of AGSLs on the cell surface, normal or treated THP-1 cells or frog peritoneal cells were collected and washed three times with cold PBS. Then, the cells were fixed with 4% paraformaldehyde for 30 mins and blocked with 3% Bovine Serum Albumin (BSA) for 1 h at room temperature (RT). Subsequently, the cells were incubated with a suitable primary antibody, such as anti-GM3 antibody (1:200 dilution) for 1 h at 37 °C because GM3 is the main ganglioside in the majority of extraneural vertebrate tissues^48^. Next, the cells were washed three times with PBS, incubated with a secondary antibody (Alexa Fluor 488-labeled donkey anti-mouse IgG/IgM κ chain, 1:200 dilution) for 30 min at 37 °C and washed three times with PBS.To detect βγ-CAT membrane binding, normal or treated THP-1 cells or frog peritoneal cells were incubated with 30 nM FITC-labeled βγ-CAT or 250 nM FITC-labeled βγ-CAT for 30 min at 37 °C and then washed three times with PBS. Finally, the cells were resuspended in 300 μl of PBS and analyzed on a flow cytometer (FACSVantage SE; Becton Dickinson, Franklin Lakes, NJ, USA). Data were analyzed using FlowJo software 7.6.1 (Tree Star Inc.).

### Western blotting

Western blotting was performed for the detection of βγ-CAT oligomerization as described previously^49^. The cells were treated with 5 nM βγ-CAT or a βγ-CAT/GSL mixture for 1 h, then the cells were lysed and separated by 12% SDS−PAGE and then electrotransferred onto polyvinylidene difluoride membranes. The membranes were subsequently blocked with 3% BSA and sequentially incubated with a rabbit anti-βγ-CAT polyclonal antibody (1:1000 dilution) and HRP-conjugated goat anti-rabbit secondary antibodies (1:5000 dilution). Finally, the protein bands were visualized with the SuperSignal WestPico chemiluminescence substrate (Invitrogen).

To detect the expression of caspase p20 and mature IL-1β in frog peritoneal cells, frog peritoneal cells were first primed with LPS (100 ng ml^−1^) for 2 h at RT and then incubated with βγ-CAT (100 nM) for 1 h at RT. Finally, the peritoneal cells were lysed for the detection of caspase p20 or βγ-CAT oligomerization by western blot, and the supernatants were concentrated to 1/10th of the original volume for the western blot analysis of mature IL-1β expression.

### BLI assay

The BLI assay was used for the biomolecule interaction study in vitro, and the detailed methods were performed as described previously^50^. In general, all interaction experiments were conducted at 37 °C in PBS (137 mM NaCl, 2.7 mM KCl, 4.3 mM Na_2_HPO_4_, 1.4 mM KH_2_PO_4_, pH 7.4) using a FortéBio Octet Red 96 instrument (FortéBio, Inc., Menlo Park, CA). For the detection of interactions between proteins and various lipids, AR2G biosensors were used. Samples (various lipids in gradient concentrations) or buffer were dispensed into solid black 96-well flat bottom plates (Greiner, Frickenhausen, GER) at a volume of 200 μl per well. The AR2G biosensors were prewet with PBS to establish a baseline before protein immobilization. Then, the different proteins (βγ-CAT, BmTFF3, MBP-αN or MBP-αC) at a concentration of 20 μg ml^−1^ were immobilized onto the AR2G biosensor according to the manufacturer’s instructions. The proteins associated with the lipids for 600 s, and the dissociation time was 1200 s. Finally, data were generated automatically by the Octet User software (version 3.1) and subsequently analyzed using Octet software version 7.0; the binding curve was globally fitted using a 1:1 model.

### Recombinant expression of MBP-αN and MBP-αC

The recombinant expression of MBP-αN and MBP-αC were performed as described previously^20^.

### Isolation of BmTFF3 and preparation of anti-BmTFF3 antibody

The BmTFF3 subunit was purified according to a previously described method with some modifications^51^. Briefly, 2.0 g of lyophilized crude skin secretions of *B*. *maxima* was separated on a DEAE Sephadex A-50 anion exchange column at pH 7.45, and the fractions were collected and further separated on a Sephadex G-50 column. Peak II of the Sephadex G-50 column was collected and applied to the AKTA Resource S cation exchange column, resulting in the separation of several protein peaks. Peak II-IV of the Resource S column was collected and finally separated with a reverse-phase HPLC Zorbax 300 SB C8 column. Peak V of the C8 column was purified BmTFF3 subunit. The purity of BmTFF3 was analyzed by SDS-PAGE with silver staining.

The preparation of a rabbit polyclonal antibody against BmTFF3 was performed as described previously^15^.

### The antibody blockade assay

To study the necessity of the BmTFF3 subunit in the βγ-CAT complex, a BmTFF3 antibody blockade assay was performed. For the inhibitory assay of βγ-CAT induced IL-1β release using anti-BmTFF3 antibody, βγ-CAT (5 nM) incubated with different concentrations of anti-BmTFF3 antibody (25, 50, 100, 200 μg ml^−1^) for 1 h at 37 °C and then added to LPS-primed THP-1 cells incubated for 2 h; the supernatant was collected for IL-1β measurement. For the blockade assay of βγ-CAT membrane binding using the anti-BmTFF3 antibody, FITC-labeled βγ-CAT (30 nM) incubated with anti-BmTFF3 antibody (100 μg ml^−1^) for 1 h at 37 °C, then added to THP-1 cells, which were incubated for 30 min at 37 °C. After washing three times with PBS, the cells were resuspended in 300 μl of PBS and analyzed by flow cytometry. For the blockade assay of βγ-CAT oligomerization using the anti-BmTFF3 antibody, βγ-CAT (5 nM) incubated with anti-BmTFF3 antibody (100 μg ml^−1^) for 1 h at 37 °C, then added to THP-1 cells, which were incubated for 30 min at 37 °C. After washing three times with PBS, the cells were lysed and subjected to western blot detection.

### Protein-lipid overlay assay

Sphingo strips (Echelon Biosciences Inc., Salt Lake City, UT) were probed with various proteins according to the manufacturer’s instructions. Briefly, the sphingo strips were blocked at RT for 2 h in blocking buffer (10 mM Tris, 150 mM NaCl, 0.1% Tween-20 and 3% BSA, pH 8.0) and washed three times with wash buffer (10 mM Tris, 150 mM NaCl and 0.1% Tween-20, pH 8.0). Purified BmTFF3 was incubated with the sphingo strips at RT for 1 h in blocking buffer at a concentration of 2 μg ml^−1^. Then, the protein solution was removed and washed three times with wash buffer. A rabbit anti-BmTFF3 polyclonal antibody (1:1000 dilution) was used as the primary antibody, and HRP-conjugated goat anti rabbit secondary antibodies (1:5000 dilution) were used as the secondary antibody. Binding was detected with the SuperSignal WestPico chemiluminescence substrate (Invitrogen).

### Isolation of lipid rafts

Lipid rafts were isolated according to the method described previously^49,52^. For the MβCD treatment, frog peritoneal cells were first treated with 5 mM MβCD for 1 h at RT and then incubated with 100 nM βγ-CAT for 30 min at RT. After washing three times with PBS, the cells were lysed and used for the isolation of lipid rafts via density gradient centrifugation. Finally, the lipid raft fractions were confirmed by western blot analysis with an anti-flotillin-1 antibody.

### Confocal microscopy

Confocal microscopy analysis was performed according to a method described previously^53^. To detect the binding of βγ-CAT and cell membrane lipid rafts or colocalization with the early βγ-CAT lysosome, frog peritoneal cells were grown on cover slips in a 24-well tissue culture plate after challenge with PMA (100 ng ml^−1^) for 12 h. Then, the adherent peritoneal cells were washed with PBS and incubated with FITC-labeled βγ-CAT (100 nM) for 1 h at 37 °C. The cells were then washed three times with PBS, fixed with 4% paraformaldehyde, and permeabilized with 0.1% Triton X-100. Next, the cells were incubated with Cy3-labeled mouse anti-flotillin-1 monoclonal antibody (1:500 dilution), FITC-labeled βγ-CAT (100 nM), mouse anti-LAMP-1 monoclonal antibody (1:50 dilution) or Cy3-labeled goat anti-mouse secondary antibody (1:500 dilution), respectively. The nuclei were stained with DAPI. Finally, the slides were observed under a confocal microscope (Olympus FV1000, Olympus Corporation, Tokyo, Japan).

### RNA interference and pharmacological inhibition assay

For the knockdown assay of key enzymes in AGSL synthesis, the shRNA lentiviral particle transduction assay was performed. THP-1 cells were plated in a 12-well plate, and adherent cells were challenged with 100 ng ml^−1^ PMA for 12 h, washed three times with PBS and cultured to 80% confluence, then removed the medium and replaced with a 1 ml polybrene/media mixture at a concentration of 5 μg ml^−1^. Next, GM3 synthase shRNA lentiviral particles, GAL3ST1 shRNA lentiviral particles or control shRNA lentiviral particles at 1.0 × 10^5^ IFU were added to the wells and incubated at 37 °C overnight. One day after transduction, the culture medium was replaced with complete medium without polybrene and cultured at 37 °C overnight. The cells expressing the shRNA were screened by puromycin dihydrochloride (5 μg ml^−1^). Finally, the cells were collected; some cells were used for determining the transduction efficiency or IL-1β release after treatment with βγ-CAT and some were used for western blot analysis.

The AGSL recovery assay was performed according to a method described previously^54^. Briefly, free AGSLs were added to the complete medium and cultured for 1 h at 37 °C. Next, the medium containing AGSLs was removed. The cells were then subjected to the detection of AGSLs expression of the cell surface by flow cytometry.

For the pharmacological inhibition of key enzymes in AGSL synthesis, PPMP, a pharmacological inhibitor of glucosylceramide synthase, the key enzyme in the biosynthesis of gangliosides, was used. Briefly, THP-1 cells were plated in a 12-well plate, challenged with PMA (100 ng ml^−1^) for 12 h, washed three times with PBS, cultured to 80% confluence, and incubated with PPMP (10 μg ml^−1^) for 28 h at 37 °C. Next, the treated cells were incubated with βγ-CAT (5 nM) for 1 h. Finally, western blotting analysis was used to detect the oligomerization of βγ-CAT, and flow cytometry analysis was used to detect the expression of gangliosides on the cell surface.

### Polymerase Chain Reaction (PCR) and real-time quantitative PCR (RT-qPCR)

The expression levels of ceramide glucosyltransferase in frog peritoneal cells were detected by PCR and RT-qPCR. Briefly, frog peritoneal cells were extracted from the peritoneal fluid of *B*. *maxima* 6 h after *A*. *hydrophila* challenge. Then, total RNA extraction and cDNA synthesis were performed according to the manufacturer’s instructions. To detect the expression of ceramide glucosyltransferase in frogs, the specific primers for PCR and RT-qPCR, listed in Supplementary Table 2, were designed using Oligo 7 software. Subsequently, 30 cycles of PCR were performed using taq polymerase. RT-qPCR was performed using the SYBR Premix Ex Taq II two-step qRT-PCR kit on a LightCycler 480 real-time PCR system (Roche LightCycler 480, Roche, Mannheim, Germany). Fold changes in the transcript levels of the target genes were analyzed by Pfaffl’s method^55^.

### In vitro frog *B*. *maxima* peritoneal cell experiments

Frog (*B*. *maxima*) peritoneal cell experiments were performed as described previously^15^. Briefly, peritoneal cells acquired from frogs and mixed to perform the subsequent experiments. For the PPMP treatment assay, the cells (4 × 10^5^ cells mL^−1^) were treated with PPMP (5, 10 and 15 μg ml^−1^) at RT for 28 h, then primed with LPS (100 ng mL^−1^) for 2 h at RT, washed twice with PBS, then the cells were treated with βγ-CAT (100 nM). Finally, the treated cells were used for flow cytometry analysis, western blotting and confocal microscopy observation as previously described^15^; the supernatants were concentrated for western blot detection of mature IL-1β.

### In vivo frog peritoneal bacterial infection assay

In vivo frog experiments were performed as described previously^15^. To assess the survival induced by PPMP, frogs were intraperitoneally injected with PPMP (100 μg kg^−1^) in 100 μl of 0.9% NaCl after anesthetization with diethyl ether. To assess the survival induced by bacteria (*A*. *hydrophila*) and the effects of PPMP on βγ-CAT, frogs were first intraperitoneally injected with PPMP (100 μg kg^−1^) in 100 μl of 0.9% NaCl for 36 h and then intraperitoneally injected with βγ-CAT (40 μg kg^−1^) 12 h before bacterial infection. Finally, the frogs were injected intraperitoneally with 1 × 10^9^ cfu of *A*. *hydrophila* to induce peritonitis. *B*. *maxima* mortality was then observed every 24 h.

For bacterial clearance, frogs were intraperitoneally injected with 1 × 10^8^ cfu of bacteria (*A*. *hydrophila*). To assess the influence of βγ-CAT on bacterial clearance, 40 μg kg^−1^ βγ-CAT was intraperitoneally injected 12 h before bacterial infection To assess the influence of PPMP on the bacterial clearance ability of βγ-CAT, PPMP (100 μg kg^−1^) was intraperitoneally injected 36 h before the intraperitoneal injection of βγ-CAT (40 μg kg^−1^) and bacteria administration under the same conditions. Finally, the number of peritoneal bacteria in all experiments at 48 h after infection was counted as described previously^15^.

### Statistical analysis

All experimental values were expressed as the mean ± SD. Each individual experiment was repeated at least two times. All the data were analyzed using GraphPad Prism 6.0 software. The survival rates of frogs were compared between groups by the Kaplan–Meier log-rank test. The significance of differences in viable bacterial counts between two groups was determined by an unpaired two-tailed Student’s *t* test. The significance of differences in IL-1β levels was determined using analysis of variance followed by an unpaired two-tailed Student’s *t-*test. The significance of differences in multi-groups comparison was determined by two-way ANOVA with Bonferroni correction. *P* <0.05 was considered statistically significant.

### Reporting Summary

Further information on experimental design is available in the Nature Research Reporting Summary linked to this article.

## Supplementary information

Supplementary Information

Supplementary Data 1

Reporting Summary

Description of Additional Supplementary Files

## Data Availability

All data supporting the findings of this study are available within the published article and its supplementary information files. The source data underlying the graphs presented in the main figures are also available in Supplementary Data 1. Additional source data or materials related to this paper may be requested from the authors.
