# Supplementary material for: Endogenous pore-forming protein complex targets acidic glycosphingolipids in lipid rafts to initiate endolysosome regulation
Source: Commun Biol. 2019 Feb 11;2:59. doi: 10.1038/s42003-019-0304-y (PMC6370762; doi:10.1038/s42003-019-0304-y)
Supplement: Supplementary file 1 — Supplementary Information [file 42003_2019_304_MOESM1_ESM.pdf]

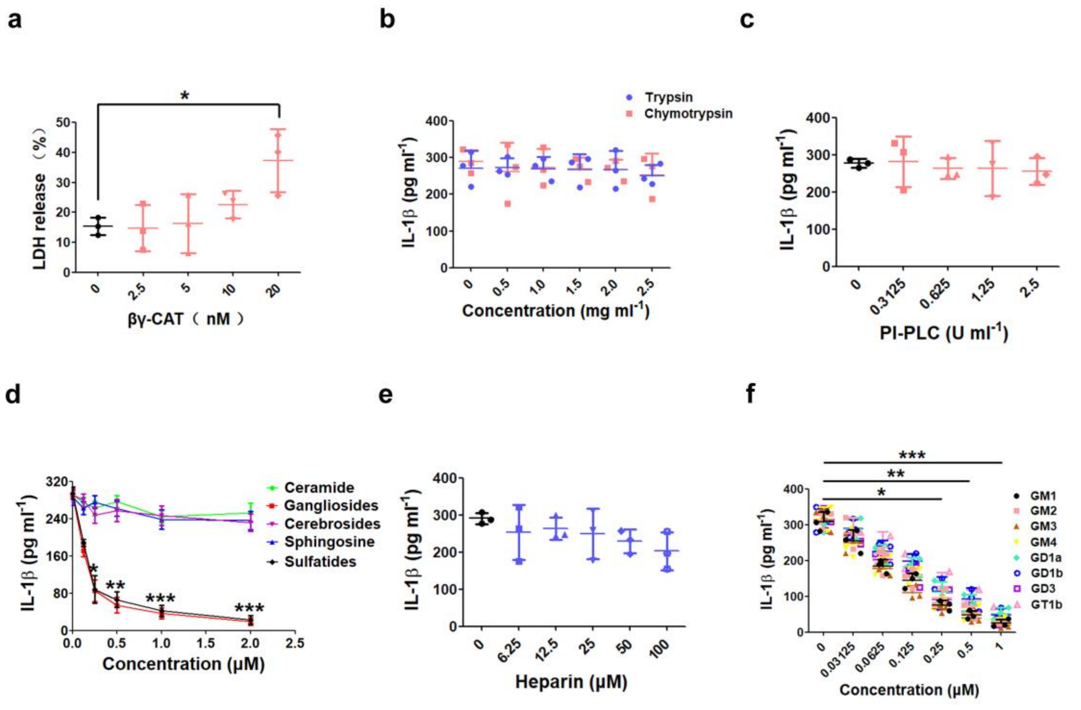

Supplementary Figure 1. The IL-1β release induced by βγ-CAT were significantly attenuated by incubating with gangliosides, but not affected after cells treated with different types of enzymes like trypsin, PI-PLC, etc. **a.** The THP-1 cells were treated with different concentrations of βγ-CAT, and cytotoxicity was determined using an LDH release assay. *$P < 0.05$ versus control by using unpaired two-tailed Student's t-test (n = 3). **b.** The LPS primed THP-1 cells were treated with a gradient concentrations of trypsin and chymotrypsin (0.5, 1.0, 1.5, 2.0 and 2.5 mg ml^{-1}), next incubating with βγ-CAT, then the IL-1β concentration in the supernatant was measured by ELISA. (n = 3). **c.** The LPS primed THP-1 cells were treated with a gradient concentrations of PI-PLC (0.3125, 0.625, 1.25 and 2.5 U ml^{-1}), next incubating with βγ-CAT, then the IL-1β concentration in the supernatant was measured by ELISA. (n = 3). **d.** βγ-CAT was incubating with different concentrations of gangliosides or other sphingolipids, next incubating with LPS-primed THP-1 cells, the IL-1β concentrations in the supernatant was measured by ELISA. *$P < 0.05$, **$P < 0.01$ and ***$P < 0.001$ represent gangliosides and sulfatides versus other lipids by two-way ANOVA with bonferroni modified (n = 3). **e.** βγ-CAT was incubating with different concentrations of

heparin, next incubating with LPS-primed THP-1 cells, the IL-1β concentrations in the supernatant was measured by ELISA. (*n* = 3). **f.** βγ-CAT was incubating with different concentrations and subtypes of ganglioside, next incubating with LPS-primed THP-1 cells, the IL-1β concentrations in the supernatant was measured by ELISA. $*P < 0.05$, $**P < 0.01$ and $***P < 0.001$ versus the respective control by unpaired two-tailed Student's t-test ($n = 3$). Bars represent the mean ± SD from three independent experiments per condition in (**a**)-(**f**). Abbreviation: PI-PLC, phosphatidylinositol-specific phospholipase C.

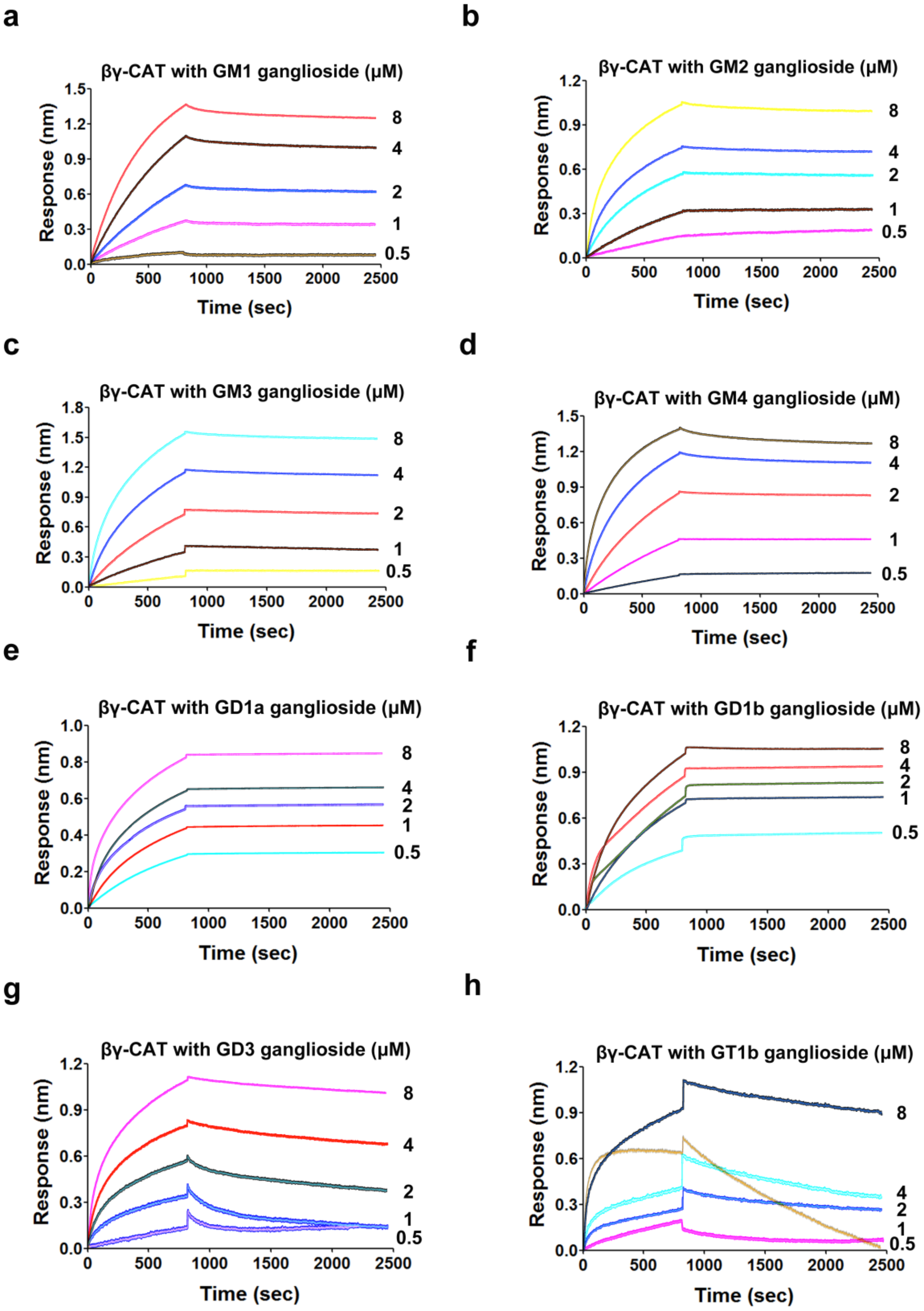
a
βγ-CAT with GM1 ganglioside (μM)
8
4
2
1
0.5
Response (nm)
Time (sec)
b
βγ-CAT with GM2 ganglioside (μM)
8
4
2
1
0.5
Response (nm)
Time (sec)
c
βγ-CAT with GM3 ganglioside (μM)
8
4
2
1
0.5
Response (nm)
Time (sec)
d
βγ-CAT with GM4 ganglioside (μM)
8
4
2
1
0.5
Response (nm)
Time (sec)
e
βγ-CAT with GD1a ganglioside (μM)
8
4
2
1
0.5
Response (nm)
Time (sec)
f
βγ-CAT with GD1b ganglioside (μM)
8
4
2
1
0.5
Response (nm)
Time (sec)
g
βγ-CAT with GD3 ganglioside (μM)
8
4
2
1
0.5
Response (nm)
Time (sec)
h
βγ-CAT with GT1b ganglioside (μM)
8
4
2
1
0.5
Response (nm)
Time (sec)

Supplementary Figure 2. The direct interaction curves between βγ-CAT and various types of gangliosides, were detected by BLI assay. **a.** The binding kinetic curves between βγ-CAT and GM1 **b.** The binding kinetic curves between βγ-CAT and GM2. **c.** The binding kinetic curves between βγ-CAT and GM3. **d.** The binding kinetic curves between βγ-CAT and GM4. **e.** The binding kinetic curves between βγ-CAT and GD1a. **f.** The binding kinetic curves between βγ-CAT and GD1b. **g.** The binding kinetic curves between βγ-CAT and GD3. **h.** The binding kinetic curves between βγ-CAT and GT1b. All BLI interaction curves (a-h) are representative of three independent experiments.

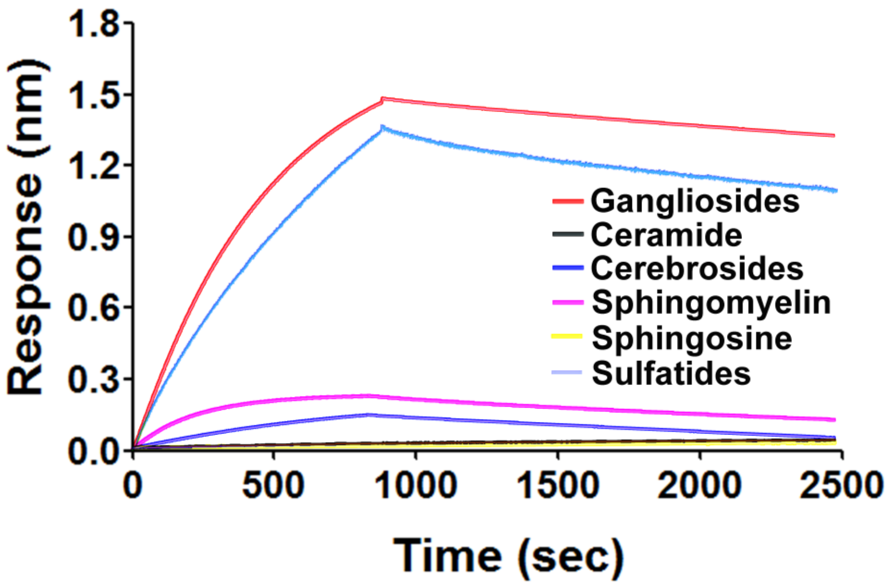

Supplementary Figure 3. βγ-CAT only interacts with AGSLs (acidic glycan contained), not other sphingolipids (without glycan or neutral glycan contained). The interaction of βγ-CAT with ceramide, sphingosine, sphingomyelin, cerebrosides and AGSLs (gangliosides or sulfatides) was detected by BLI assay. The BLI interaction curves are representative of three independent experiments.

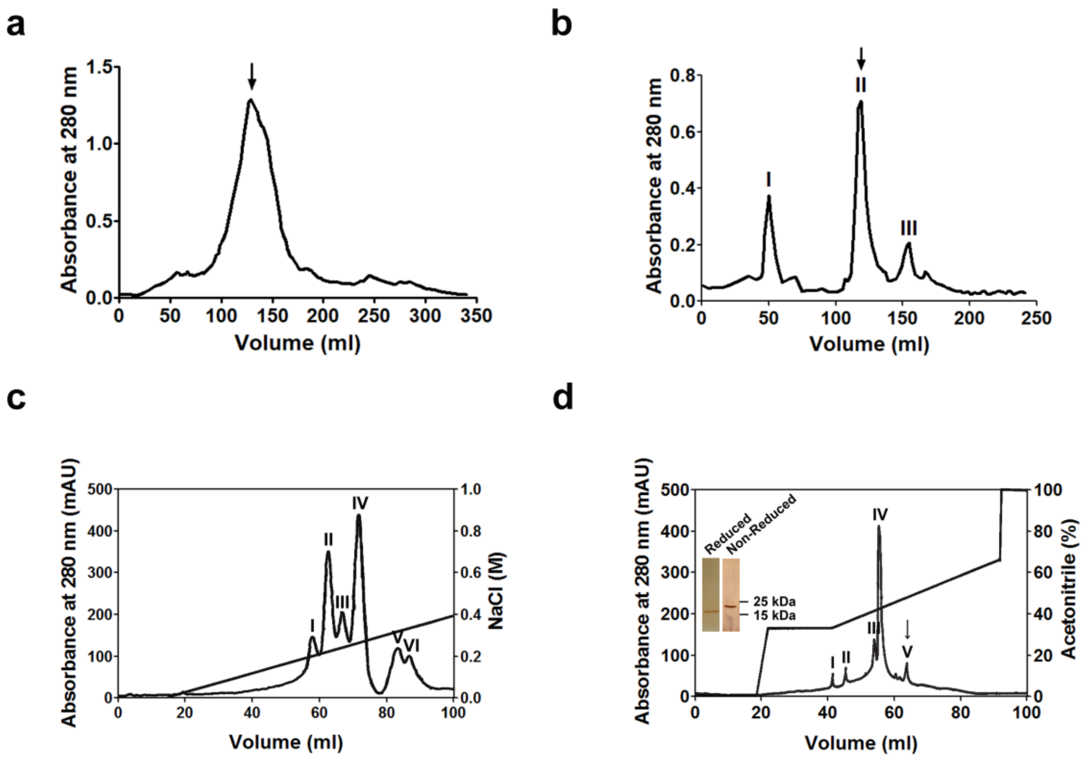

Supplementary Figure 4. The purification and identification of BmTFF3 from the skin secretions of *B.maxima*. **a.** The isolation and purification of BmTFF3 from the skin secretions of frog was performed by using DEAE Sephadex A-50. **b.** The Sephadex G-50 isolation curve of BmTFF3 after isolated by DEAE Sephadex A-50. **c.** The isolation curve of Resource S cation exchange column of peak II after isolated by Sephadex G-50. **d.** The peak II-IV of Resource S column was isolated by using the HPLC C8 column, the peak V was demonstrated as the purified BmTFF3 by mass spectrum and western blotting (data not shown).The purity of peak V was identified by SDS-PAGE and silver staining. The inserted gel graph represented the silver staining results of peak V of HPLC C8 in SDS-PAGE under reduced and non-reduced conditions.

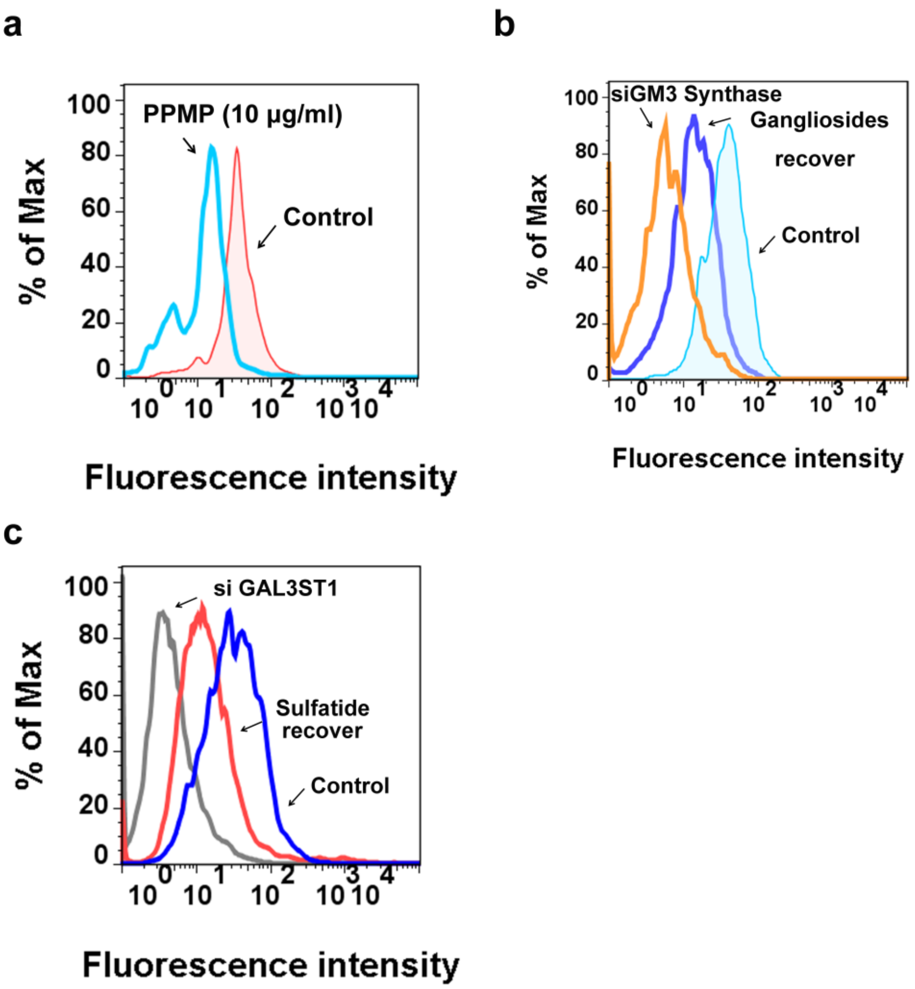

Supplementary Figure 5. The expression of gangliosides or sulfatides on the cell surface of THP-1 cells after the key enzyme of gangliosides or sulfatides synthesis blocked by PPMP or knocked down by RNAi. **a.** The THP-1 cells was treated with PPMP (10 μg ml^{-1}) at 37 °C for 28 h, then the contents of cell surface gangliosides was detected by flow cytometry. The PPMP untreated THP-1 cells as control. **b.** Knock-down the ganglioside expression of THP-1 cells by transfected with GM3 Synthase shRNA lentivirus and re-addition of free gangliosides to the knock-down THP-1 cells, the ganglioside contents of THP-1 cells was detected by flow cytometry. The untreated normal THP-1 cells as control. **c.** Knock-down the expression of GAL3ST1 of THP-1 cells by transfected with GAL3ST1 shRNA lentivirus and re-addition of free sulfatides to the knock-down THP-1 cells, the sulfatide contents of THP-1 cells was detected by flow cytometry. The untreated normal THP-1 cells as control.

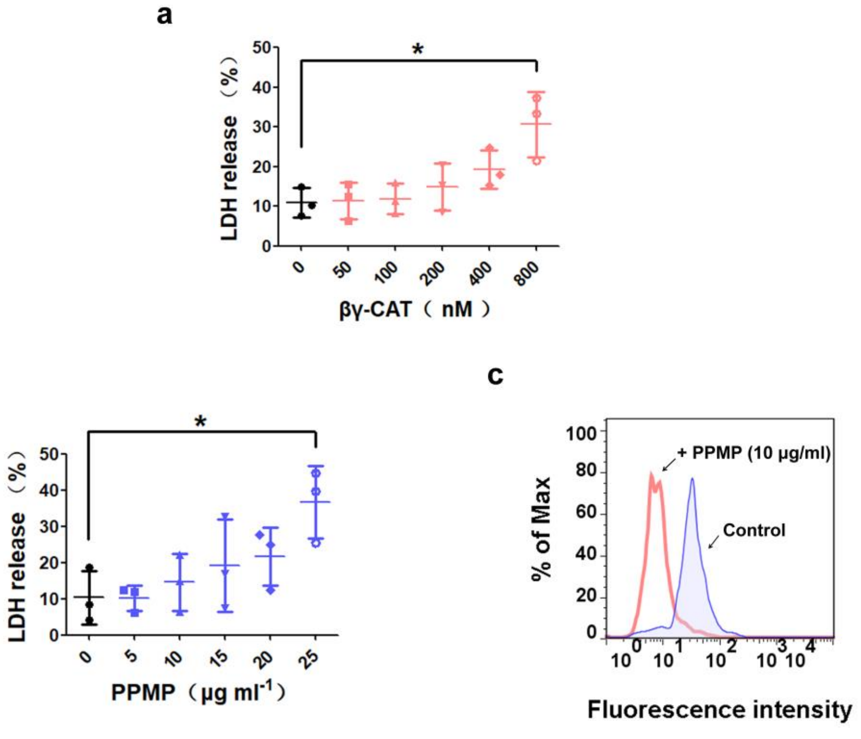

Supplementary Figure 6. The cytotoxicity assay of frog peritoneal cells treated with βγ-CAT or PPMP. **a.** The frog peritoneal cells were treated with different concentrations of βγ-CAT for 2 h, and cytotoxicity was determined using an LDH release assay. *$P < 0.05$ versus control by using unpaired two-tailed Student's t-test (n = 3). **b.** The frog peritoneal cells were treated with different concentrations of PPMP at room temperature for 28 h, then the LDH of supernatant was detected by using the LDH release detection kit. *$P < 0.05$ versus control by using unpaired two-tailed Student's t-test (n = 3). **c.** The ganglioside expression of cell surface of frog peritoneal cells were detected by flow cytometry after the cells treated with 10 μg ml^{-1} PPMP. The PPMP untreated frog peritoneal cells as control. Bars represent the mean ± SD from three independent experiments per condition in (**a**) and (**b**).

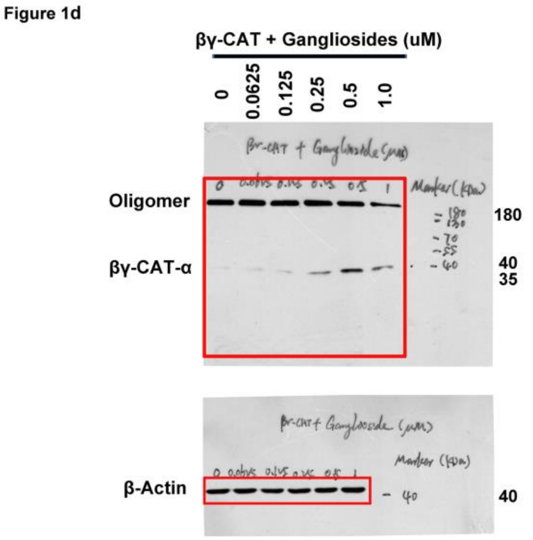

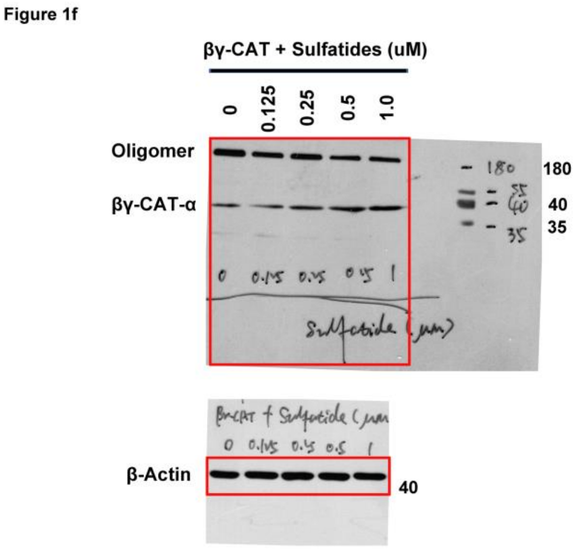

Supplementary Figure 7. Original immunoblots for Fig. 1.

Figure 2g

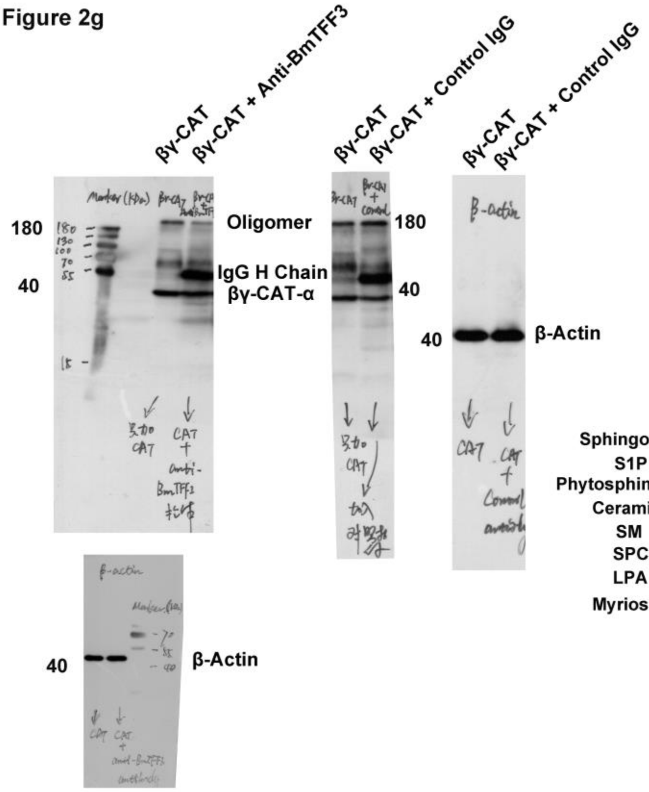

Figure 2h

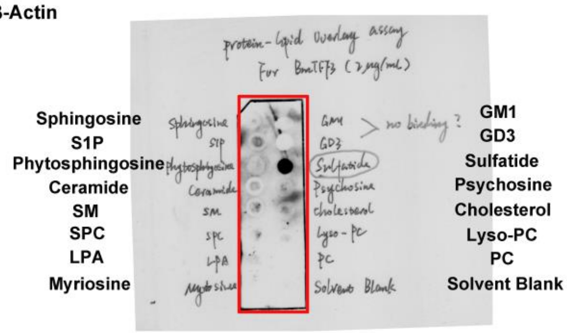

Supplementary Figure 8. Original immunoblots for Fig. 2.

Figure 3a

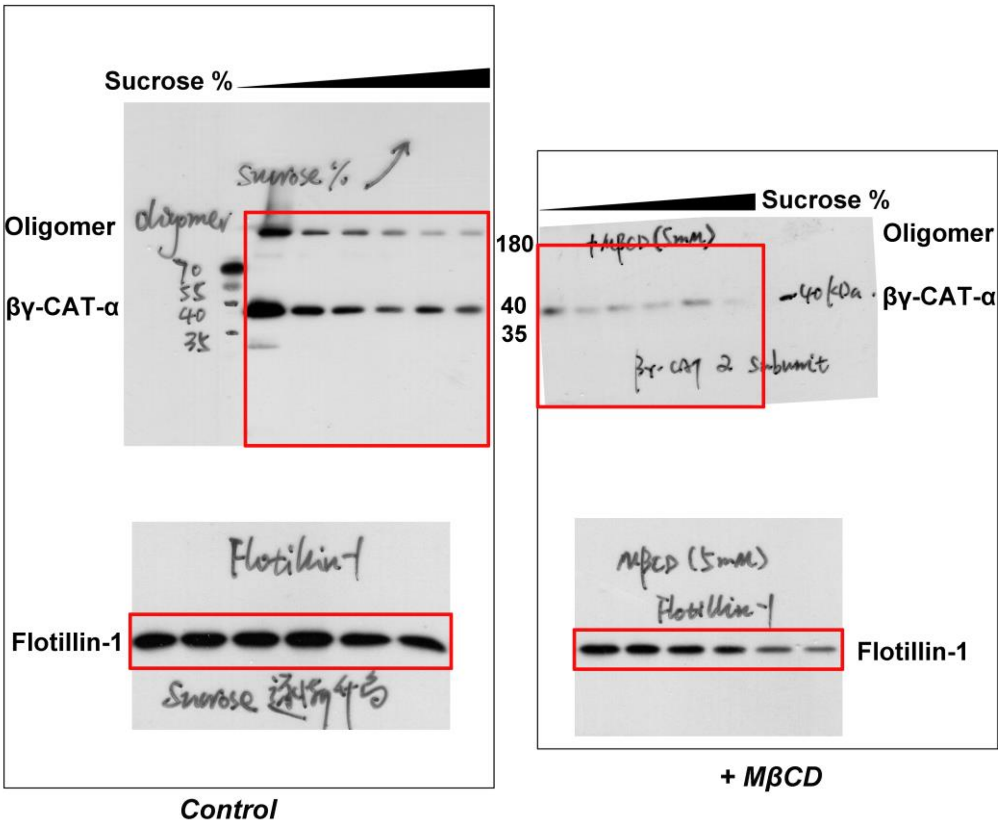

Supplementary Figure 9. Original immunoblots for Fig. 3a.

Figure 3d

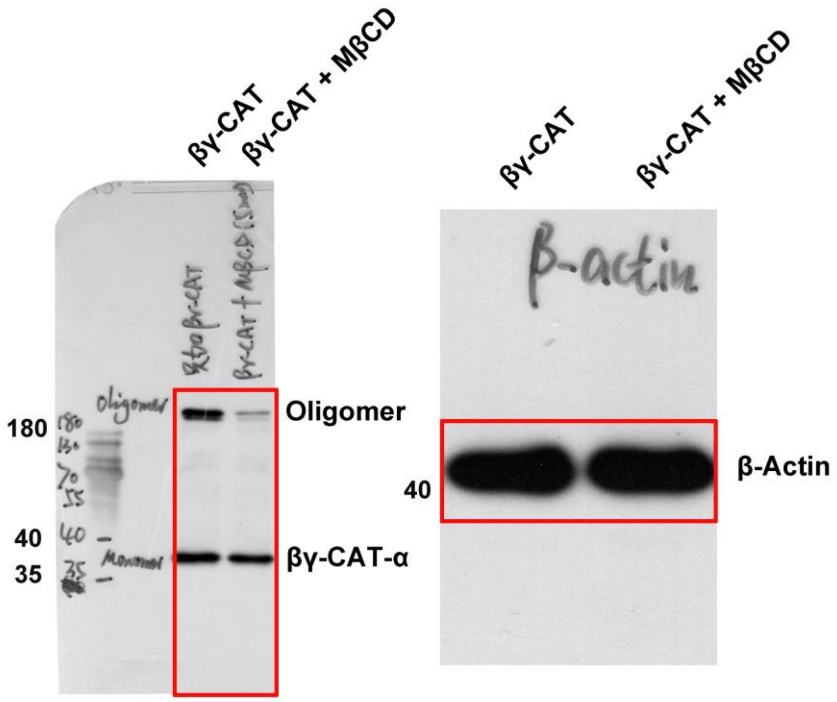

Supplementary Figure 10. Original immunoblots for Fig. 3d.

Figure 3e

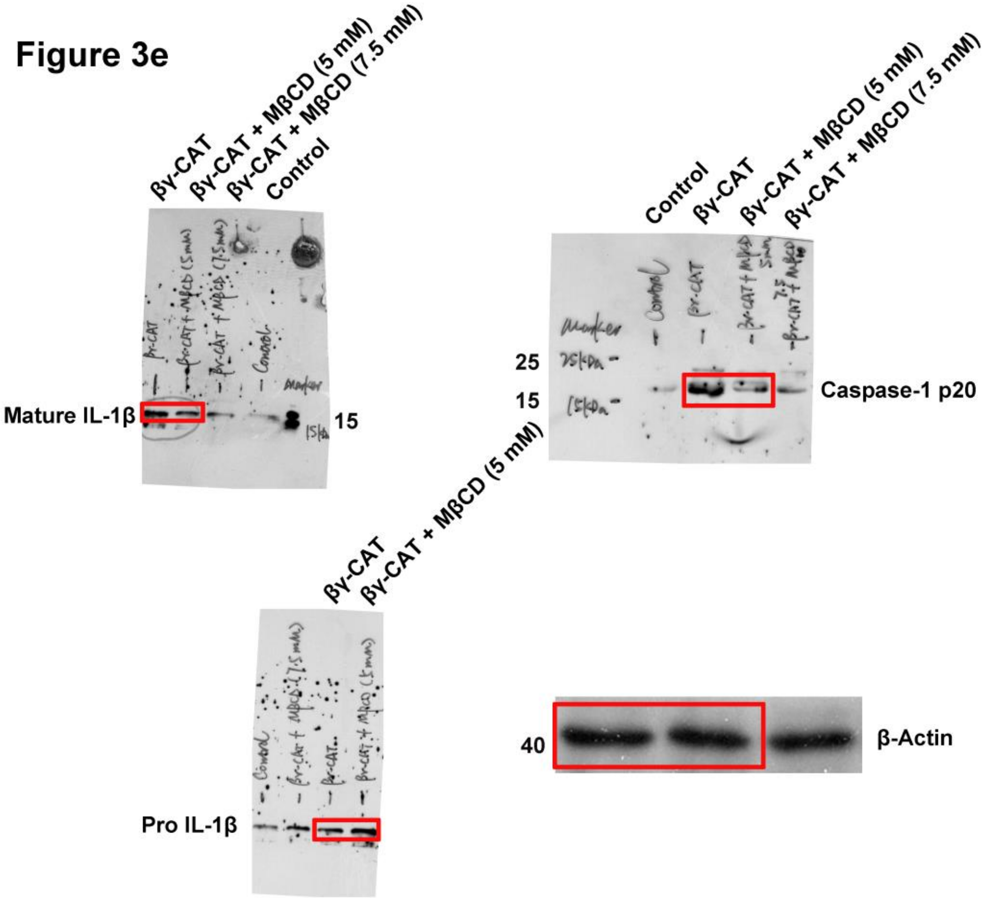

Supplementary Figure 11. Original immunoblots for Fig. 3e.

Figure 4b

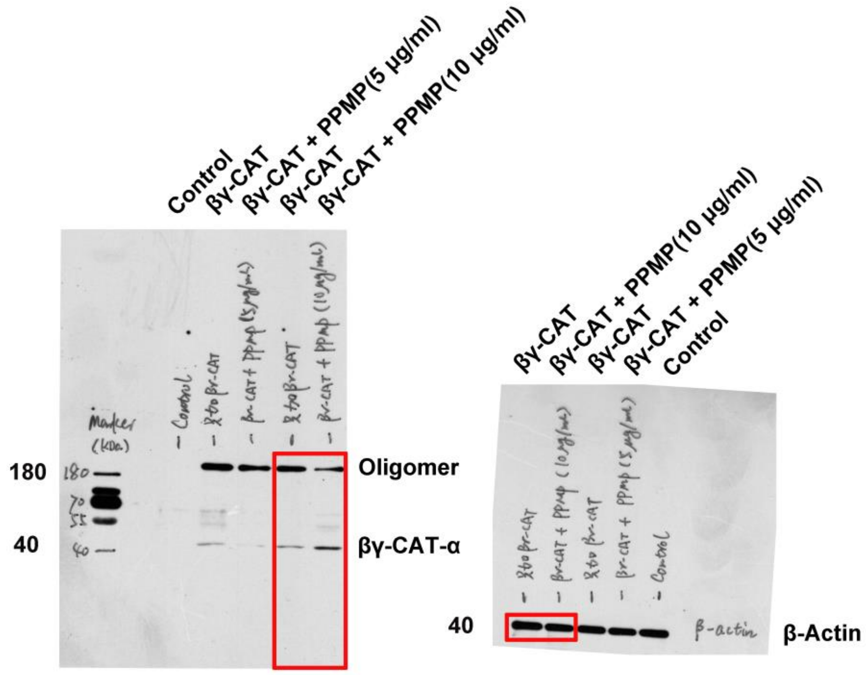

Supplementary Figure 12. Original immunoblots for Fig. 4b.

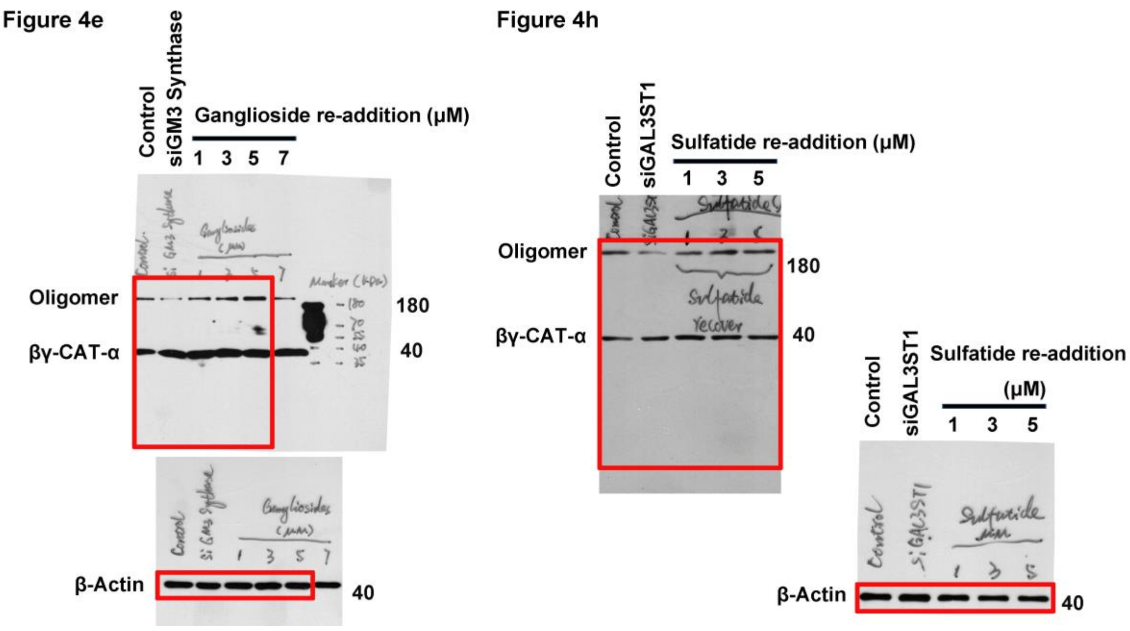

Supplementary Figure 13. Original immunoblots for Fig. 4e and Fig. 4h.

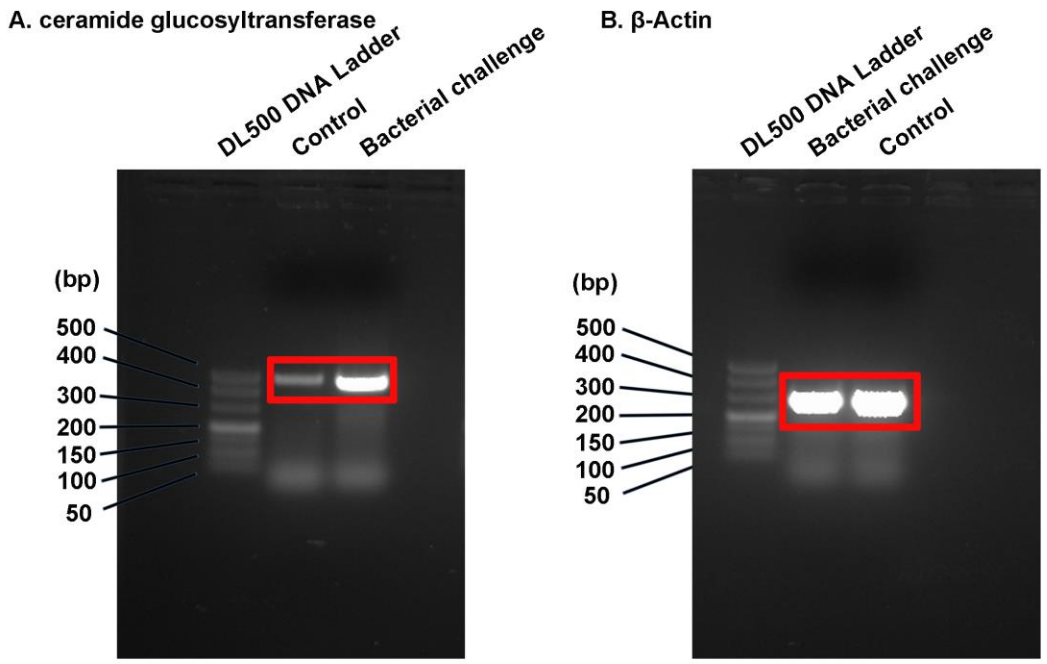

Supplementary Figure 14. Original gels for Fig. 5a.

Figure 5f

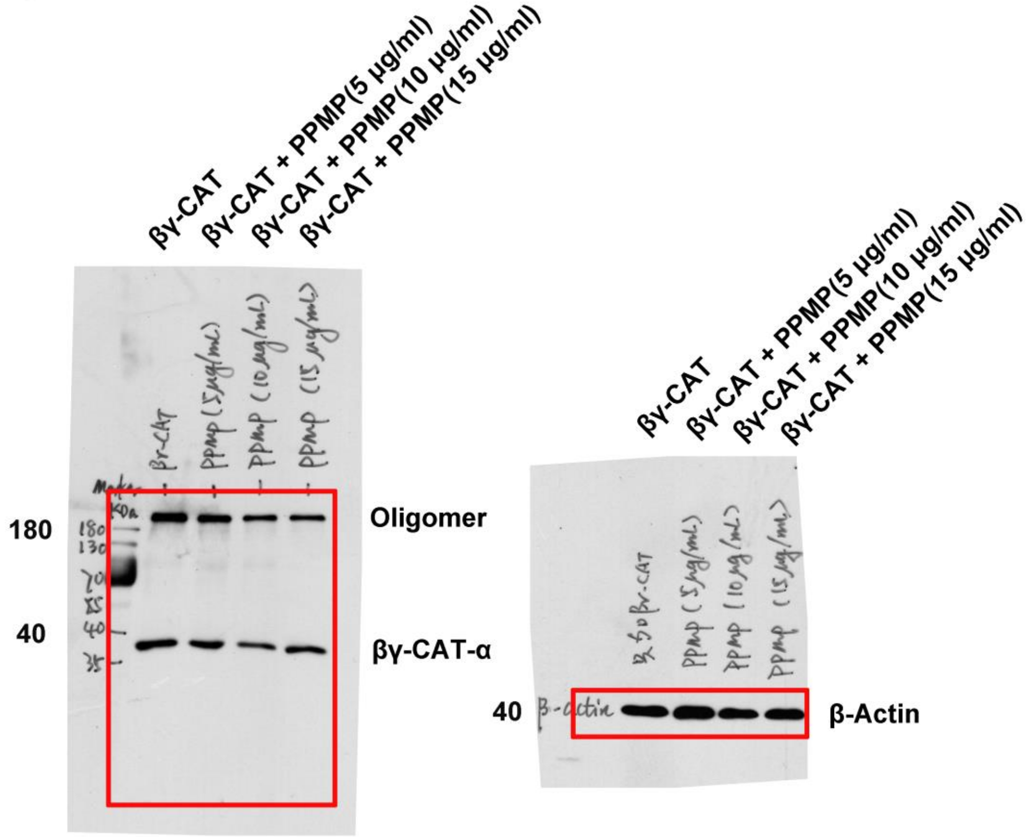

Supplementary Figure 15. Original immunoblots for Fig. 5f.

Figure 5g

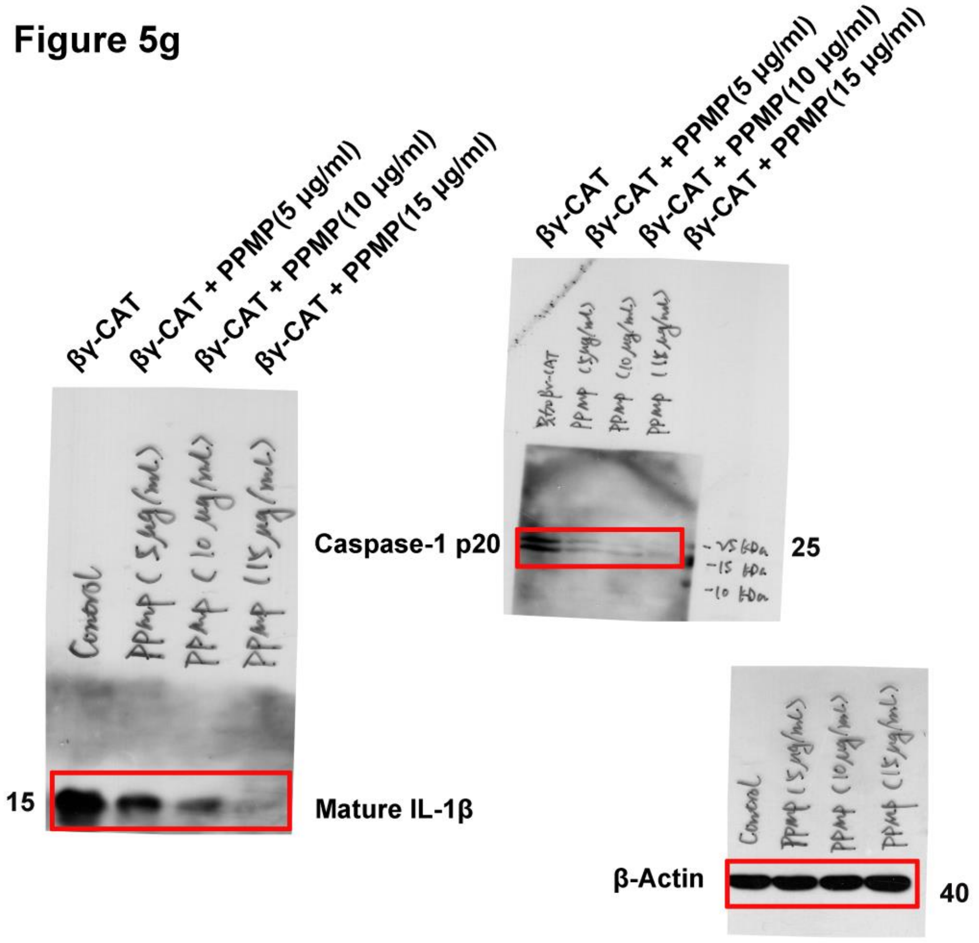

Supplementary Figure 16. Original immunoblots for Fig. 5g.

Supplementary Table 1. The calculated K_D value between βγ-CAT and different types of gangliosides.

**Ganglioside subtypes**	***K_D* value with βγ-CAT**
GM1	(3.05 ± 0.18) x 10^{-8} M
GM2	(5.37 ± 0.23) x 10^{-8} M
GM3	(1.02 ± 0.11) x 10^{-8} M
GM4	(4.63 ± 0.33) x 10^{-8} M
GD1a	(7.63 ± 0.38) x 10^{-8} M
GD1b	(5.21 ± 0.42) x 10^{-7} M
GD3	(4.67 ± 0.36) x 10^{-8} M
GT1b	(5.27 ± 0.47) x 10^{-7} M

Supplementary Table 2. Sequences of primers used for detection of ceramide glucosyltransferase of frog *B. maxima*.

**Primer**	**Sequence**	**Product length**
**Semi-quantitative PCR**		
β-actin-Forward	GTAGCCCCTGAAGAACACCC	238 bp
β-actin-Reverse	TTGCATGGGGCAGAGCATAA	
Ceramide glucosyltransferase		
Forward	CAGGTGGACTTATTGCCTTTGCTCA	474 bp
Ceramide glucosyltransferase		
Reverse	AGTCAAATATGAACCACGCTAAGCAG	
**Real-time quantitative PCR**		
β-actin-Forward	GTAGCCCCTGAAGAACACCC	238 bp
β-actin-Reverse	TTGCATGGGGCAGAGCATAA	
Ceramide glucosyltransferase		
Forward	CAGGTGGACTTATTGCCTTTGCTCA	171 bp
Ceramide glucosyltransferase		
Reverse	TTAGCTTGGCCCACCTGATCATCCG	
