# Supplementary material for: Endogenous pore-forming protein complex targets acidic glycosphingolipids in lipid rafts to initiate endolysosome regulation
Source: Commun Biol. 2019 Feb 11;2:59. doi: 10.1038/s42003-019-0304-y (PMC6370762; doi:10.1038/s42003-019-0304-y)
Supplement: Supplementary file 3 — Reporting Summary [file 42003_2019_304_MOESM3_ESM.pdf]

nature research

Corresponding author(s): Yun Zhang

# Reporting Summary

Nature Research wishes to improve the reproducibility of the work that we publish. This form provides structure for consistency and transparency in reporting. For further information on Nature Research policies, see Authors & Referees and the Editorial Policy Checklist.

## Statistical parameters

When statistical analyses are reported, confirm that the following items are present in the relevant location (e.g. figure legend, table legend, main text, or Methods section).

| n/a | Confirmed | |
|---|---|---|
| ☐ | ☒ | The exact sample size ($n$) for each experimental group/condition, given as a discrete number and unit of measurement |
| ☐ | ☒ | An indication of whether measurements were taken from distinct samples or whether the same sample was measured repeatedly |
| ☐ | ☒ | The statistical test(s) used AND whether they are one- or two-sided<br>*Only common tests should be described solely by name; describe more complex techniques in the Methods section.* |
| ☐ | ☒ | A description of all covariates tested |
| ☐ | ☒ | A description of any assumptions or corrections, such as tests of normality and adjustment for multiple comparisons |
| ☐ | ☒ | A full description of the statistics including central tendency (e.g. means) or other basic estimates (e.g. regression coefficient) AND variation (e.g. standard deviation) or associated estimates of uncertainty (e.g. confidence intervals) |
| ☐ | ☒ | For null hypothesis testing, the test statistic (e.g. $F$, $t$, $r$) with confidence intervals, effect sizes, degrees of freedom and $P$ value noted<br>*Give P values as exact values whenever suitable.* |
| ☐ | ☒ | For Bayesian analysis, information on the choice of priors and Markov chain Monte Carlo settings |
| ☐ | ☒ | For hierarchical and complex designs, identification of the appropriate level for tests and full reporting of outcomes |
| ☐ | ☒ | Estimates of effect sizes (e.g. Cohen's $d$, Pearson's $r$), indicating how they were calculated |
| ☐ | ☒ | Clearly defined error bars<br>*State explicitly what error bars represent (e.g. SD, SE, CI)* |

*Our web collection on statistics for biologists may be useful.*

## Software and code

Policy information about availability of computer code

| | |
|---|---|
| Data collection | We state that no software was used in our study. |
| Data analysis | We state that no software was used in our study. |

For manuscripts utilizing custom algorithms or software that are central to the research but not yet described in published literature, software must be made available to editors/reviewers upon request. We strongly encourage code deposition in a community repository (e.g. GitHub). See the Nature Research guidelines for submitting code & software for further information.

## Data

Policy information about availability of data

All manuscripts must include a data availability statement. This statement should provide the following information, where applicable:

- Accession codes, unique identifiers, or web links for publicly available datasets
- A list of figures that have associated raw data
- A description of any restrictions on data availability

Data availability: All data needed to evaluate the conclusions in the paper are present in the paper and/or the Supplementary Materials. Additional data related to this paper may be requested from the authors.
The figures that have associated raw data are listed below:

Fig 1C, right;
Fig 1D, right;
Fig 2D, right;
Fig 2E;
Fig 3A;
Fig 3D;
Fig 3E;
Fig 4B;
Fig 4E;
Fig 4H;
Fig 4I;
Fig 5A, left;
Fig 5E;
Fig 5F

# Field-specific reporting

Please select the best fit for your research. If you are not sure, read the appropriate sections before making your selection.

☒ Life sciences ☐ Behavioural & social sciences ☐ Ecological, evolutionary & environmental sciences

For a reference copy of the document with all sections, see nature.com/authors/policies/ReportingSummary-flat.pdf

# Life sciences study design

All studies must disclose on these points even when the disclosure is negative.

| | |
|---|---|
| Sample size | In our study, the size of samples (such as frogs, peritoneal cells) were determined based on our previous study. For example, the frogs used in our study are commonly weight of 25 ± 5 g , each group should be contain at least 5 frogs. For the size of cells, different number of cells were used in different assays, but we did at least duplicate in parallel. |
| Data exclusions | There is no data was excluded before statistical analysis. |
| Replication | All of our assays are done at least three independent experiments and could be replicated successfully, such as the interaction detection by using fortebio octet system, western blot analysis of the oligomerization of βγ-CAT, RNAi assay of the key enzyme in glycosphingolipid synthesis and the recover assay of glycosphingolipids, etc. |
| Randomization | In our study, the samples (such as frogs) were allocated into experimental groups completely random. |
| Blinding | Yes, we were blinded to group allocation during data collection and following analysis. |

# Reporting for specific materials, systems and methods

## Materials & experimental systems

| n/a | Involved in the study | |
|---|---|---|
| ☒ | ☐ | Unique biological materials |
| ☐ | ☒ | Antibodies |
| ☐ | ☒ | Eukaryotic cell lines |
| ☒ | ☐ | Palaeontology |
| ☐ | ☒ | Animals and other organisms |
| ☒ | ☐ | Human research participants |

## Methods

| n/a | Involved in the study | |
|---|---|---|
| ☒ | ☐ | ChIP-seq |
| ☐ | ☒ | Flow cytometry |
| ☒ | ☐ | MRI-based neuroimaging |

## Antibodies

| | |
|---|---|
| Antibodies used | The antibodies used in our study were listed as below:<br>1. βγ-CAT full-length rabbit polyclonal antibody and anti-BmTFF3 rabbit polyclonal antibody were prepared by our laboratory.<br>2. Frog IL-1beta rabbit polyclonal antibody and frog caspase-1 p20 rabbit polyclonal antibody were prepared by our laboratory.<br>3. Beta Actin Mouse Monoclonal antibody, Catalog number: 60008-1-Ig, CloneNo.: 7D2C10, was purchased from Proteintech Group, Inc.<br>4. Flotillin 1 Rabbit Polyclonal antibody, Catalog number: 15571-1-AP, was purchased from Proteintech Group, Inc.<br>5. Anti-monosialoganglioside GM3 monoclonal antibody (M2590) was purchased from Cosmo Bio Co, LTD. |

6. Anti-O4 monoclonal antibody (O7139) was purchased from Sigma-Aldrich.
7. Anti-GM3 Synthase antibody (B-12) and anti-GAL3ST1 antibody (sc-86462) were purchased from Santa Cruz Biotechnology.
8. HRP conjugated Goat anti-mouse IgG (H+L), Catalog number: SA00001-1, was purchased from Proteintech Group, Inc.
9. HRP conjugated Goat Anti-Rabbit IgG （H+L） Secondary Antibody (BA1054) was purchased from BOSTER.
10. Alexa Fluor 488 conjugated Goat anti-Mouse IgG / IgM (H+L) secondary antibody was purchased from Thermo Fisher Scientific Inc.

Validation

All of the above primary antibodies were testified valid in our study. The detailed description as below:
1. βγ-CAT full-length rabbit polyclonal antibody, species: Bombina maxima, application: WB, IP and IF.
2. Anti-BmTFF3 rabbit polyclonal antibody, species: Bombina maxima, application: WB, IP and IF.
3. Anti IL-1β and anti-caspase-1 p20 rabbit polyclonal antibody, species: Bombina maxima, application: WB, IP and IF.
4. Beta-actin mouse monoclonal antibody, species: human, mouse, rat, pig, plant, et al, application: WB, IHC, IF, FC, ELISA.
5. Flotillin 1 Antibody, species: human, mouse, rat , application: WB, IP, IHC, IF, FC, ELISA.
6. Anti-GM3 monoclonal antibody, species: This antibody recognize GM3(NeuAc) and cross-react with Sialylpara globoside(NeuAc) and GM3 Lactone.
7. Monoclonal anti-oligodendrocyte marker O4 antibody produced in mouse has been used in immunocytochemistry and immunofluorescence.
8. The GM3 Synthase antibody (B-12), recommended for detection of GM3 Synthase of mouse, rat and human origin by WB, IP, IF and ELISA.

# Eukaryotic cell lines

Policy information about cell lines

Cell line source(s)

THP-1 cells (ATCC® TIB-202™) was purchased from ATCC.

Authentication

The THP-1 cells we used in our study was authenticated by Kunming Cell Bank of Type Culture Collection, CAS.

Mycoplasma contamination

The THP-1 cells we used in our study was tested negative for mycoplasma contamination.

Commonly misidentified lines
(See ICLAC register)

There is no any misidentified cell line with THP-1 can be found in Database of Cross-Contaminated or Misidentified Cell Lines.

# Animals and other organisms

Policy information about studies involving animals; ARRIVE guidelines recommended for reporting animal research

Laboratory animals

We state that our study did not involve laboratory animals.

Wild animals

We state that our study did not involve wild animals.

Field-collected samples

In our study, the frog Bombina maxima (adult male or female) as experimental animal. Frogs (B. maxima) with a mean body weight of 25 ± 5 g were caught in their natural environment, then they were placed into tanks filled with dechlorinated tap water between 20 °C and 24 °C for 1 wk before the experiments. These tanks were equipped with four platforms, and four animals were placed into each tank. The frogs were fed live Tenebrio molitor.

# Flow Cytometry

## Plots

Confirm that:

☒ The axis labels state the marker and fluorochrome used (e.g. CD4-FITC).

☒ The axis scales are clearly visible. Include numbers along axes only for bottom left plot of group (a 'group' is an analysis of identical markers).

☒ All plots are contour plots with outliers or pseudocolor plots.

☒ A numerical value for number of cells or percentage (with statistics) is provided.

## Methodology

Sample preparation

The normal or treated THP-1 cells or frog peritoneal cells were collected, washed three times with cold PBS, then the cells fixed with 4% paraformaldehyde for 30 mins and blocked with 3% BSA for 1 h at RT, subsequently the cells incubated with mouse anti-GM3 antibody (1:200 diluted) since GM3 was the main ganglioside in the majority of extraneural tissues of vertebrata 48 for 1 h at 37 ℃. Next the cells washed three times with PBS and incubated with secondary antibody (Alex 488 labeled donkey anti mouse IgG/IgM κ chain, 1:200 diluted) for 30 min at 37°C, then washed three times with PBS, discard the supernatant and the cells resuspended with 300 μL PBS, the resuspended cells were analyzed with a flow cytometer.
For the membrane binding detection of βγ-CAT, the normal or treated THP-1 cells or frog peritoneal cells were incubated with 30 nM FITC labelled βγ-CAT or for 30 min at 37°C, then washed three times with PBS. Finally, the cells were resuspended with 300 μL PBS and analyzed with a flow cytometer.

Instrument

FACSVantage SE (Becton Dickinson, Franklin Lakes, NJ, USA).

| | |
|---|---|
| Software | FlowJosoftware 7.6.1 (Tree Star Inc). |
| Cell population abundance | The abundance of cell population was mainly depended on the forward scatter (FSC) and side scatter (SSC) of THP-1 cells or frog peritoneal cells. For THP-1 cells, the cell population was single and could be thought of as a group of pure cells. |
| Gating strategy | The gating we used was depended on the positive and negative control of our assay. |

☐ Tick this box to confirm that a figure exemplifying the gating strategy is provided in the Supplementary Information.
