# Supplementary material for: Endogenous pore-forming protein complex targets acidic glycosphingolipids in lipid rafts to initiate endolysosome regulation
Source: Commun Biol. 2019 Feb 11;2:59. doi: 10.1038/s42003-019-0304-y (PMC6370762; doi:10.1038/s42003-019-0304-y)
Supplement: Supplementary file 4 — Description of Additional Supplementary Files [file 42003_2019_304_MOESM4_ESM.pdf]

Description of Additional Supplementary Files

File Name: Supplementary Data 1
Description: The source data of all dot-plot graphs and line graphs in the manuscript.
